# Translocations can drive expression changes of multiple genes in regulons covering entire chromosome arms

**DOI:** 10.1093/nar/gkaf677

**Published:** 2025-08-22

**Authors:** Anna Oncins, Roser Zaurin, Houyem Toukabri, Kimberly Quililan, José R Hernández Mora, Magdalena A Karpinska, Erik Wernersson, Alastair Smith, Agostina Bianchi, Leone Albinati, Luca Cozzuto, Andrea Rivero, Chloe Gulliver, Jessica Velten, Johanna Denkena, François Serra, Raúl Gómez, Cristina López, Sílvia Beà, Jonas Paulsen, Nadia Halidi, Alfonso Valencia, Magda Bienko, A Marieke Oudelaar, Renée Beekman

**Affiliations:** Centre for Genomic Regulation (CRG), Barcelona Institute of Science and Technology (BIST), C. Dr. Aiguader 88, Barcelona 08003, Spain; Department of Medicine and Life Sciences, Universitat Pompeu Fabra (UPF), Barcelona 08003, Spain; Centre for Genomic Regulation (CRG), Barcelona Institute of Science and Technology (BIST), C. Dr. Aiguader 88, Barcelona 08003, Spain; Centre for Genomic Regulation (CRG), Barcelona Institute of Science and Technology (BIST), C. Dr. Aiguader 88, Barcelona 08003, Spain; Centre for Genomic Regulation (CRG), Barcelona Institute of Science and Technology (BIST), C. Dr. Aiguader 88, Barcelona 08003, Spain; Centre for Genomic Regulation (CRG), Barcelona Institute of Science and Technology (BIST), C. Dr. Aiguader 88, Barcelona 08003, Spain; Department of Genome Organization and Regulation, Max Planck Institute for Multidisciplinary Sciences, Göttingen 37077, Germany; Faculty of Biology and Psychology, Georg August University of Göttingen, Göttingen 37073, Germany; Department of Microbiology, Tumor and Cell Biology, Karolinska Institutet, Stockholm 17165, Sweden; Department of Cell and Molecular Biology, Science for Life Laboratory, Solna 17165, Sweden; MRC Molecular Haematology Unit, MRC Weatherall Institute of Molecular Medicine, University of Oxford, Oxford OX3 9DS, UK; MRC WIMM Centre for Computational Biology, MRC Weatherall Institute of Molecular Medicine, University of Oxford, Oxford OX3 9DS, UK; Centre for Genomic Regulation (CRG), Barcelona Institute of Science and Technology (BIST), C. Dr. Aiguader 88, Barcelona 08003, Spain; Department of Medicine and Life Sciences, Universitat Pompeu Fabra (UPF), Barcelona 08003, Spain; Centre for Genomic Regulation (CRG), Barcelona Institute of Science and Technology (BIST), C. Dr. Aiguader 88, Barcelona 08003, Spain; Department of Medicine and Life Sciences, Universitat Pompeu Fabra (UPF), Barcelona 08003, Spain; Centre for Genomic Regulation (CRG), Barcelona Institute of Science and Technology (BIST), C. Dr. Aiguader 88, Barcelona 08003, Spain; Centre for Genomic Regulation (CRG), Barcelona Institute of Science and Technology (BIST), C. Dr. Aiguader 88, Barcelona 08003, Spain; Centre for Genomic Regulation (CRG), Barcelona Institute of Science and Technology (BIST), C. Dr. Aiguader 88, Barcelona 08003, Spain; Centre for Genomic Regulation (CRG), Barcelona Institute of Science and Technology (BIST), C. Dr. Aiguader 88, Barcelona 08003, Spain; Centre for Genomic Regulation (CRG), Barcelona Institute of Science and Technology (BIST), C. Dr. Aiguader 88, Barcelona 08003, Spain; Life Sciences Department, Barcelona Supercomputing Center, Barcelona 08034, Spain; Present: Carlos Simon Foundation, 46980 Paterna, Valencia, Spain; Centre for Genomic Regulation (CRG), Barcelona Institute of Science and Technology (BIST), C. Dr. Aiguader 88, Barcelona 08003, Spain; Lymphoid Neoplasms Program, Institut d’Investigacions Biomèdiques August Pi i Sunyer (IDIBAPS), Barcelona 08036, Spain; Departamento de Tumores Hematológicos, Centro de Investigación Biomédica en Red de Cáncer (CIBERONC), Madrid 28029, Spain; Departament de Fonaments Clínics, University of Barcelona, Barcelona 08036, Spain; Hematopathology Section, Pathology Department, Hospital Clínic Barcelona, Barcelona 08036, Spain; Lymphoid Neoplasms Program, Institut d’Investigacions Biomèdiques August Pi i Sunyer (IDIBAPS), Barcelona 08036, Spain; Departamento de Tumores Hematológicos, Centro de Investigación Biomédica en Red de Cáncer (CIBERONC), Madrid 28029, Spain; Departament de Fonaments Clínics, University of Barcelona, Barcelona 08036, Spain; Hematopathology Section, Pathology Department, Hospital Clínic Barcelona, Barcelona 08036, Spain; Department of Biosciences, Faculty of Mathematics and Natural Sciences, University of Oslo, 0316 Oslo, Norway; Centre for Genomic Regulation (CRG), Barcelona Institute of Science and Technology (BIST), C. Dr. Aiguader 88, Barcelona 08003, Spain; Life Sciences Department, Barcelona Supercomputing Center, Barcelona 08034, Spain; Department of Life & Medical Sciences, Institució Catalana de Recerca Avançada (ICREA), Barcelona 08010, Spain; Department of Microbiology, Tumor and Cell Biology, Karolinska Institutet, Stockholm 17165, Sweden; Department of Cell and Molecular Biology, Science for Life Laboratory, Solna 17165, Sweden; Human Technopole, Viale Rita Levi-Montalcini 1, 20157 Milan, Italy; Department of Genome Organization and Regulation, Max Planck Institute for Multidisciplinary Sciences, Göttingen 37077, Germany; Centre for Genomic Regulation (CRG), Barcelona Institute of Science and Technology (BIST), C. Dr. Aiguader 88, Barcelona 08003, Spain; Department of Medicine and Life Sciences, Universitat Pompeu Fabra (UPF), Barcelona 08003, Spain; Lymphoid Neoplasms Program, Institut d’Investigacions Biomèdiques August Pi i Sunyer (IDIBAPS), Barcelona 08036, Spain

## Abstract

Chromosomal translocations have largely been implicated in tumor development. However, beyond the consequences of aberrant gene expression near the breakpoint, their effects remain underexplored. In this work, we characterize the interplay between translocations, chromatin organization and gene expression using mantle cell lymphoma (MCL) as a model. We show by *in vitro* genomic engineering and in MCL patient samples that translocations can drive transcriptional changes at entire chromosome arms affecting multiple genes in a regulon-like fashion. Moreover, we demonstrate a clear link between the translocation-induced transcriptional alterations and genome organization, with genes most susceptible to change expression forming pre-existing ultra-long-range interactions spanning 50 megabases. The translocation involves the strong immunoglobulin enhancer into this 3D interaction, allowing the spread of its regulatory potential over the entire affected chromosome arm. Finally, we show that translocation-induced effects mainly represent expression enhancement of genes already active prior to translocation formation, highlighting the importance of the epigenetic state of the cell in which this initial hit occurs. In summary, by studying genome organization principles in the context of translocations, we describe a new principle of gene regulation, showing that strong enhancers can induce substantial gene expression enhancement through ultra-long-range interactions affecting entire chromosome arms, representing an important new mechanism in health and disease.

## Graphical Abstract

**Graphical Abstract:**
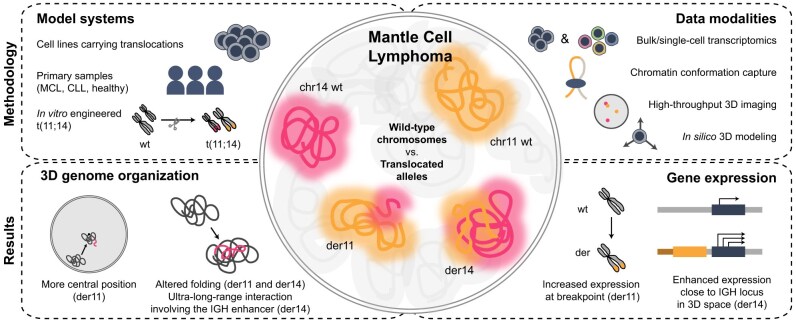

## Introduction

With the advancement of chromosome conformation capture (3C) and next-generation sequencing technologies, our knowledge of the cell's nuclear structure has evolved drastically. It is now widely accepted that the large intricacy of mammalian organisms cannot only be explained by the linear genomic sequence [1, 2]. Instead, it is widely established that mammalian nuclei are highly structured organelles at the three-dimensional (3D) level, exhibiting multiple scales of complexity [3]. From promoter-enhancer loops [4] to topologically associating domains (TADs) [5] and A and B compartments [6], the genome is organized in a non-random fashion, contributing to gene expression regulation and cell diversity. At the largest scale beyond these intrachromosomal genome organization characteristics, a clear level of interchromosomal organization exists, whereby chromosomes occupy separate spaces, known as chromosome territories (CT) that intermingle little with each other [7]. Recent studies have furthermore shown a correlation between the radial positioning of CTs and certain chromosomal characteristics, such as length, gene density, and GC content [8, 9], indicating that the genome is also highly organized at the interchromosomal level.

Structural variants (SVs) including deletions, duplications, inversions, insertions and translocations, lead to global changes in the genome, not only affecting its linear sequence but also its 3D organization at all the aforementioned levels. While structural genomic rearrangements contribute to genetic diversity in eukaryotes, they can also play a key role in disease development and progression. With respect to tumor formation, since the description of the translocation t(9;22)(q34.1;q11.2), also known as the Philadelphia chromosome, in chronic myeloid leukemia (CML) in the 1960s [10, 11], SVs have been recurrently associated with malignant transformation. The t(15;17)(22q;q12) translocation was for example discovered in acute promyelocytic leukemia (APL) patients [12]. First attempts to understand the oncogenic effect of these SVs revealed the formation of chimeric or fusion proteins such as BCR::ABL and PML::RAR, generated as a direct consequence of the translocation in CML and APL, respectively [12, 13]. Later, new evidence showed that the effect of SVs could also rely on the formation of new intrachromosomal interactions, driving gene expression alterations beyond fusion proteins. Following efforts focused on understanding SV-induced enhancer hijacking and enhancer activation loss, for example at the *EVI1/GATA2* locus in acute myeloid leukemias (AML) carrying inversions or translocations of chromosome 3 [14]. Yet, the effect of SVs, and translocations in particular, at the long-range intrachromosomal and interchromosomal level remains less investigated. It was not until the last decade that the first evidence linking SVs with global genome reorganization was provided. For instance, studies carried out on Ewing's sarcoma patients demonstrated a significant relocalization of translocated chromosomes 11 and 22 within the cell nucleus [15, 16]. More recently, the description of systematic changes of contact frequencies between short and long chromosomes in the presence of aneuploidies, such as in Patau syndrome (trisomy of chromosome 13) or Edwards syndrome (trisomy of chromosome 18), confirmed the link between SVs and nuclear reorganization [17]. Yet, the mechanisms by which SVs affect nuclear architecture and which functional consequences these changes have remain largely unclear.

In this context, non-Hodgkin lymphomas (NHLs) represent a good model to study the effect of SVs on tumor progression, since many cases harbor translocations involving the IGH enhancer locus and different proto-oncogenes [18]. In this study, we specifically focus on mantle cell lymphoma (MCL), a lymphoid malignancy that accounts for 5–10% of all NHLs [19–21]. MCLs frequently display the t(11;14)(q13;q32) translocation, which leads to the juxtaposition of the IGH enhancer next to the *CCND1* proto-oncogene, a positive regulator of cell cycle. Nevertheless, CCND1 overexpression alone is not sufficient to drive tumor formation [22, 23], suggesting that the effect of the translocation could be broader than previously appreciated. Recent advances in chromatin organization have shed light on how enhancer-promoter interactions and A-B compartments are altered in MCL [24]. However, the effect of translocations on the global interchromosomal interaction landscape, as well as on the long-range intrachromosomal 3D organization in MCL, remains largely unclear. Additionally, the consequences of these changes for lymphoma initiation and progression are still unknown. Therefore, in this study we aimed to explore the intricate interplay between translocations, chromatin architecture and gene expression, using MCL as a model. To that end, we combined 3C, *in silico* 3D genome modeling, and 3D fluorescence *in situ* hybridization (FISH) data from MCL patient samples and/or cell lines—allele-specific where possible—with gene expression analysis in MCL patient samples and upon *in vitro* translocation generation. Through these complementary analyses, we show that translocated chromosomes can undergo extensive genome organization alterations that are tightly linked to large-scale gene expression changes. More specifically, we describe that IGH translocations can drive transcriptomic alterations of multiple genes covering entire chromosome arms, turning these long genomic segments into large regulatory units. Interestingly, the overexpressed genes in our *in vitro* translocation-induced models and MCL patients are enriched at the exact same genomic regions, underlining the potential relevance of our finding for lymphomagenesis. Moreover, we show that pre-existing chromatin interactions are exploited by translocations, allowing the regulatory effect of strong enhancers to spread over more than 50 megabases. We furthermore demonstrate that the translocation-induced effects mainly represent an enhanced expression of genes already active prior to translocation formation. Overall, these results highlight the importance of the epigenetic state of the tumor cell of origin in which an initial hit occurs. In summary, our findings can have a major impact on understanding the role of ultra-long-range interactions in shaping the gene expression landscape in physiological and pathophysiological conditions, such as early tumor formation, representing an important new mechanism in health and disease.

## Materials and methods

### Samples and cell lines

#### Primary samples

No primary samples were processed for experiments in this study. All Hi-C and RNA-seq data of patient samples and healthy donors were mined from publicly available data from a previous publication [24]. Sample characteristics can be found in Supplementary Table S1.

#### Cell lines

Cell lines Z-138, JVM2 and Pfeiffer were obtained from ATCC (cat. no. CRL-3001; cat. no. CLR-3002; cat. no. CRL-2632), U266 from DSMZ (cat. no. ACC 9), KARPAS422 from Merck (cat. no. CB_06 101 702), and GM12878 from Coriell (cat. no. CVCL_7526). Z-138 cells were cultured in Iscove’s Modified Dulbecco’s Medium containing 4 mM L-glutamine (Gibco^TM^, cat. no. 31 980 030), supplemented with 10% horse serum (Gibo^TM^, cat. no. 10 368 902) and 1% penicillin/streptomycin (Gibco^TM^, cat. no. 15 140 122). JVM2, U266 and GM12878 cells, were cultured in RPMI-1640 Medium 2 mM L-glutamine (Gibco^TM^, cat. no. 21 875 034) containing 15% fetal bovine serum (FBS) (Gibco^TM^, cat. no. 10 270 106) and 1% penicillin/streptomycin (Gibco^TM^, cat. no. 15 140 122). KARPAS422 and Pfeiffer cells were cultured in RPMI-1640 medium with 20% and 10% FBS, respectively. All cells were cultured at 37°C under 5% CO_2_ and atmospheric O_2_ conditions, and they were regularly passed and tested for mycoplasma infection.

### Bivariate flow karyotyping, chromosome sorting and sequencing

Z-138 cells were prepared for flow karyotyping as described [25] with few modifications. Briefly, 20 million cells were treated with 0.1 μg/ml Colcemid (Sigma-Aldrich, cat. no. 4 725 301) for 6 h at 37°C. Pelleted cells were resuspended in 10 ml hypotonic solution (75 mM KCl, 10 mM MgSO4, 0.2 nM spermine and 0.5 nM spermidine, pH 8.0) and incubated for 27 min at 37°C. After centrifugation (5 min at 300 g), the cell pellet was resuspended in 1 ml cold polyamine solution buffer (15 mM Tris pH 8.0, 2 mM EDTA, 0.5 mM EGTA, 80 mM KCl, 3 mM DTT, 0.25% Triton X-the chromosomes. Next, samples were vigorously vortexed for 30 s and filtered through a 35 μm mesh filter (Falcon, cat. no. 352 235). Chromosomes were double stained by adding 10 μl of 1M MgSO_4_.7H_2_O, 40 μg/ml of chromomycin A3 (Sigma-Aldrich, cat. no. C2659), an AT-bp specific dye, and 5 μg/ml of Hoechst 33 258 (Sigma-Aldrich, cat. no. H3569), a CG-bp specific dye, for 8 h at 4°C. Before sorting, samples were incubated with 10 mM of sodium citrate and 25 mM of sodium sulfite for 2 h on ice. Flow karyotyping for chromosome sorting was performed on the BD Influx cell sorter (Becton Dickinson, San Jose, CA) using the UV (355 nm) and Deep Blue (457 nm) high laser power and 100 μm nozzle. Sorted chromosomes were treated with 200 μg/ml of RNAse (NEB, cat. no. EN0531) for 30 min at 37°C and 200 μg/ml with Proteinase K (Invitrogen, cat. no. AM2546) for 16 h at 50°C. After treatment, the buffer was exchanged by dialysis using Pur-A-Lyzer™ Maxi Dialysis Kit (Sigma-Aldrich, cat. no. PURX50005) against 1x TE buffer for 48 h at RT. To reduce the volume, samples were concentrated by evaporation in a SpeedVac Vacuum concentrator up to 100 μl volume and purified with 2x SPRI beads (Beckman Coulter™ Agencourt AMPure XP, cat. no. A63880). Sequencing libraries were prepared using the NEBNext Ultra II FS DNA Library Prep Kit (NEB, cat. no. E7805). Libraries of around 300 bp size were sequenced using the Illumina HiSeq 2500 system (paired-end, 2 × 125 bp). Libraries corresponding to the wild-type chromosomes 9 to 12 and der11 were sequenced for a total of 133 and 43.4 million reads, respectively.

### Hi-C data generation

Hi-C was performed on Z-138 as previously described [26] with minor modifications reported here. Briefly, 30 million cells were cross-linked with 1% formaldehyde (Merck, cat. no. F8775-4 × 25ML), quenched with 0.125 M glycine solution (Sigma-Aldrich, cat. no. 50 046) and flash-frozen in dry ice. Samples were then stored at -80°C until further steps were performed. After lysis, chromatin was digested using two restriction enzyme conditions, with two or three biological replicates per condition. Cells were either digested with 72 μl MboI (25 U/μl, NEB, cat. no. R0147M), or a mix of three restriction enzymes: 30 μl of NcoI-HF (20 U/μl, NEB, cat. no. R3193S), 30 μl of BclI-HF (20 U/μl, NEB, cat. no. R3160S) and 30 μl BsrGI-HF (20 U/μl, NEB, cat. no. R3575S) and incubated overnight at 37°C without shaking. All the enzymes were added at two different time-points to refresh their enzymatic activity, except for BsrGI, which was only added once, as its enzymatic activity lasts longer than 8 h. Next, enzymes were heat-inactivated for 20 min at 65°C (MboI) or 80°C (combination of three restriction enzymes). Finally, chromatin was extracted using the ethanol extraction method. The final concentration was calculated by Qubit (dsDNA High Sensitivity Assay) and ligation and digestion efficiency were checked on a 0.6% agarose gel.

Hi-C libraries were built from 4 μg of DNA per sample and sonicated using a BioruptorPico device (Diagenode, cat. no. B01060010, 3 cycles, 20 s on, and 90 s off). The efficiency of sonication was checked on a 1.2% agarose gel. Sheared samples were cleaned up using 1/3x pre-washed Streptavidin T1 beads (T1 Dynabeads MyOne, Invitrogen, cat. no. 65 601), incubated for 30 min in 100 μl of the end-repair library mix (10 μl of 1x T4 DNA ligase buffer with 10 mM ATP (10x, NEB, cat. no. B0202S), 2 μl of dNTPs (25 mM each), 5 μl of T4 PNK (10 000 U/ml, NEB, cat. no. M0201S), 4 μl of T4 DNA polymerase (3 000 U/ml, NEB, cat. no. M0203S) and 1 μl of the DNA polymerase I large (Klenow) fragment (NEB, cat. no. M0210L)), and reclaimed with 100 μl of A-tailing mix (10 μl of 10x NEBuffer2; 5 μl of 10 mM dATP; and 5 μl of the Exo minus Klenow fragment (3′ 5′), 5000 U/ml, NEB, cat. no. M0212S) for 30 min at 37°C. Illumina loop adaptors (2.5 μl, NEBNext Multiplex Oligos for Illumina, Index primer set 1, NEB, cat. no. E7335S) were ligated to the DNA fragments in 50 μl of the Quick ligation mix containing 25 μl of 2x Quick Ligase reaction buffer (NEB, cat. no. B2200S) and 2 μl of T4 DNA ligase (2 000 000 U/ml, NEB, cat. no. M2200M), for 15 min at RT with 3 μl of the USER adaptor (NEBNext Multiplex Oligos for Illumina, NEB, cat. no. E7338A) for an additional 15 min at 37°C. Final libraries were resuspended in 10 μl of EB buffer (10 mM Tris-Cl, pH 8) from Qiagen (cat. no. 19 086) and amplified using 2.5 μl of Universal PCR primer (NEBNext Multiplex Oligos for Illumina, NEB, cat. no. E6861A), 2.5 μl of indexed primers (NEBNext Multiplex Oligos for Illumina, Index primer set 1, NEB, cat. no. E7335), and the NEBNext High Fidelity PCR Master Mix (12.5 μl of 2x master mix, NEB, cat. no. M0541S), during eight PCR cycles (10 s of denaturalization at 98°C, 30 s of annealing at 65°C and 30 s of elongation at 72°C). The final libraries were extracted using 70% EtOH and resuspended in 30 μl EB buffer, quantified by Qubit (dsDNA High Sensitivity Assay) and profiles were evaluated on the Bioanalyzer 2100. Libraries were then sequenced on a HiSeq2500 (50 bp, paired-end reads, 60 million reads per library on average).

### Hi-C and bivariate flow karyotype data processing and allele-specific Hi-C analysis

#### Hi-C data processing

Hi-C data of 5 individual MCLs, 7 individual CLLs, 12 individual normal B-cells, and 5 Z-138 replicates were processed. Hi-C reads were iteratively mapped using TADbit mapper [27] against genome build GRCh38. Mapped reads were parsed and duplicated reads or reads with a distance of less than 1 Kbp between the two read pairs were filtered out. Valid pairs were then normalized using TADbit norm at two different resolutions, 100 Kb and 1 Mb (‘Vanilla’ normalization, >1000 reads per bin). Biases files (created by the TADbit norm command) and bam files with valid pairs (created by the TADbit mapper command) were used for further processing. First, after obtaining raw contact frequency matrices per individual primary sample using the TADbit bin function at 1 Mb resolution, these were normalized to a total interaction count of 150 M per sample. Next, these matrices were merged (using the median interaction frequency per interaction bin) to obtain three merged raw contact matrices, one for MCL (*n* = 5), one for CLL (*n* = 7), and one for normal B cells (*n* = 12). Furthermore, compartments were assigned per sample using the TADbit segment function (using the 100 Kbp resolution data). Per chromosome and per sample, the eigenvectors representing the compartment scores were defined by selecting the one with a high correlation (∼0.6 or higher) between the eigenvalues and GC content. In case the correlation with GC content was similar for multiple eigenvectors and/or when the difference in the percent of variation explained by different eigenvectors was less than 1.5 fold, visual inspection was conducted on Pearson's heatmaps of the eigenvectors. To obtain one compartment score per 1 Mbp bin in each individual sample, the median compartment scores of ten 100 Kbp bins were defined. Finally, to obtain one compartment score per sample type (MCL, CLL, or normal B cells), the median compartment scores were calculated to merge the individual 1 Mbp scores. A and B compartments were assigned having a positive or negative eigenvalue, respectively.

#### Interchromosomal interaction frequencies in primary samples

First of all, to avoid artifacts from regions with low-mappability, interactions from outlier 1 Mb bins with <60.000 interactions in total were flagged to be ignored (set to NA—non-applicable). These mainly represent telomeric and centromeric regions. Next, to obtain pairwise interchromosomal interaction frequencies between chromosomes in MCL, CLL, and normal B cells, we calculated the total interaction counts of the merged matrices per chromosome pair and divided this number by the product of the total number of Mb bins per chromosome (scaling the interaction frequency per square Mbp bin). Log2FC of the interaction frequencies was calculated by dividing the obtained values in MCL by the values obtained in MCL or CLL.

#### Bivariate flow karyotype data analysis Z-138

Reads of the sorted chromosomes were trimmed using TrimGalore (version 0.6.6), with a minimal Phred quality score of 13, a maximal allowed error rate of 0.1, a stringency of 3 and a minimal read length of 35 bp. Quality checks of the reads before and after trimming were carried out by fastQC (version 0.11.9). Next, trimmed reads were aligned against the reference GRCh38 human genome using hisat2 (version 2.2.1), with default parameters, a minimal allowed intron length of 20 bp and a maximal of 500 000 bp. Then, genotype calling was carried out following the variant discovery workflow present in the GATK Best Practices recommendations (https://gatk.broadinstitute.org/hc/en-us/sections/360007226651-Best-Practices-Workflows). After marking duplicates ('MarkDuplicatesSpark'), the base quality scores were recalibrated first by producing the recalibration table ('BaseRecalibrator' using the known polymorphic sites from the GRCh38 104 human genome release version) and then by applying the recalibration ('ApplyBQSR') to the original BAM files with the aligned reads. Variants were then called independently for each chromosome sorting sample, limiting the calling to chromosomes 11 and 14, and using 'HaplotypeCaller' in GVCF mode. Next, these per-sample variants were joined creating a database with all the consolidated per-sample variants ('GenomicsDBImport') and the joined genotyping with ‘GenotypeGVCFs.’ All these arguments were launched with default arguments. The used GATK version was 4.2.0.0, and its associated Picard version was 2.25.0. Based on the bivariate flow karyotype data we also assigned the chromosomal breakpoint regions in Z-138, resulting in the following assignments in this cell line: chr11 up < 69 582 000, chr11 down > 69 583 000, chr14 up < 105 890 000, and 14 down > 105 891 000 Mb.

#### Heterozygous SNP assignments and allele-specific genotype calls in Z-138

Heterozygous SNPs on chromosomes 11 and 14 in Z-138 were determined using a combination of SNP array, bivariate flow karyotype, and Hi-C data. The latter two were generated in this study, while the former data were produced in the context of a previous publication [28]. The SNP array data of two replicates of Z-138 was analyzed using the *crlmm* R package (v. 1.58.0). Default quality scores for SNPs (>0.7) and samples (>5) were used as filtering thresholds. SNPs assigned as heterozygous in both replicates were considered further. Similarly to the processing of the fastQ files of the bivariate flow karyotype data described above, Hi-C data was processed to obtain heterozygous SNP calls. Bam files of the five replicates were merged after base recalibration followed by variant calling in GVCF mode and selection of heterozygous SNPs with a quality score above the default threshold in GATK. Only for chr11 up, heterozygous SNPs could also be determined using the bivariate flow karyotype data. To that end, genetic variants with a genotype quality score equal to or greater than 20 were used to retrieve the genotype information of the wild-type chromosome 11 and the der11. By comparing the obtained genotypes, positions with heterozygous SNPs were defined. Next, for each heterozygous SNP on chromosomes 11 and 14, the genetic variants with a genotype quality score equal to or greater than 20 of the chromosome sorting data were used to assign a specific genotype call to derivatives 11 and 14 (Supplementary Table S2).

#### Allele-specific Hi-C analysis Z-138

The Hi-C read sequences covering heterozygous SNP with allele-specific genotype calls were consequently extracted from the merged bam file, containing the reads of all 5 Z-138 Hi-C replicates. Using Bedtools (version v2.27.1) intersect, the BAM files were screened for overlaps with the heterozygous SNP positions, whereby only reads with at least one overlap were kept. Next, the base calling of the heterozygous SNP position was retrieved from the read sequences. Alleles were assigned to the wild-type or derivative chromosome by comparing the SNP base pair of the read with the assigned reference and alternate base calls. Finally, the number of interactions from the wild-type and translocated fragments of chromosomes 14 and 11 to each chromosome was calculated.

### 4C-seq data generation and analysis

#### Sample preparation

4C-seq was performed as previously described with minor modifications [29]. Briefly, 10 million cells were crosslinked with 2% formaldehyde (Merck, cat. no. F8775-4 × 25ML), prior to quenching with 0.125 M glycine solution (Sigma-Aldrich, cat. no. 50 046) and lysis for 10 min in ice-cold lysis buffer (50 mM Tris–HCl pH 7.5, 150 mM NaCl, 5 mM EDTA, 0.5% NP-40, 1% TX-100) supplemented with 1X cOmplete EDTA-free Protease Inhibitor (Roche; cat. no. 04 693 132 001). Cell pellets were flash-frozen on dry ice and stored short term at -80°C until first digestion. Cell pellets were then resuspended in 1x DpnII buffer and three replicates per condition were digested with 500 U DpnII (NEB; 50 000 U/mL; cat. no. R0543M) at 37°C and 900 RPM, at three subsequent times to refresh enzymatic activity (4 h, overnight, 4 h again). DpnII was heat-inactivated for 20 min at 65°C, prior to ligation (T4 DNA Ligase; Roche; cat. no. 10 716 359 001) and PCI/ethanol precipitation, as previously described [29]. DNA pellets were resuspended in 10 mM Tris–HCl pH 7.5 and stored at -20°C. Cells were then resuspended in 1X NEBuffer r2.1 (NEB; cat. no. B6002S) and digested overnight with 100 U Csp6I (NEB; 10 000 U/mL; #R0639S) at 25°C, 900 RPM, prior to PCI/ethanol precipitation (as Csp6I cannot be heat-inactivated). Second ligation and final PCI/ethanol precipitation were then performed as previously described [29]. Each digestion and ligation efficiency stage were checked on 0.6% agarose gels at each step, and final DNA concentration was calculated by Qubit dsDNA High-Sensitivity assay kit (Thermo Scientific; cat. no. Q32854). PCR amplification of the 4C DNA template was performed using the Expand Long Template PCR System (Sigma-Aldrich; cat. no. 11 681 842 001) using 200 ng 4C template per reaction and viewpoint specific primers (fw and rv primer sequences for CCND1: ACTCCAGCAGGGCTTCGATC & GTATTTGCATAACCCTGAGC; YAP1: AGAAATGATACTCATGGATC & GGAGGAATATTTGTGTGAAAG; CD3: CACACAGACTAGGGCAGATC & TGGGAGTTGAGTATAAGGACA; fw and rv adapter sequences: AATGATACGGCGACCACCGAGATCTACACTCTTTCCCTACACGACGCTCTTCCGATCT & CAAGCAGAAGACGGCATACGAGAT/index/GTGACTGGAGTTCAGACGTGTGCTCTTCCGATCT, with index representing a 6 bp index sequence). For GM12878 and Z138, 16 PCR reactions were performed for all viewpoints, whereas for the *in vitro* translocation model sample, 16 reactions performed for the CCND1 viewpoint, and 10 reactions for CD3 and YAP1 viewpoints. Samples were then pooled and purified using the High Pure PCR Product Purification Kit (Sigma-Aldrich; cat. no. 11 732 676 001).

#### Sequencing library preparation

PCR products were checked by the Bioanalyzer 2100 (Aligent, using the Agilent High Sensitivity DNA Kit, cat. no. 5067–4626 and Agilent High Sensitivity DNA Reagents, cat. no. 5067–4627). Next, samples were pooled in equimolar ratios and sequencing libraries were built using the Ligation Sequencing Kit V14 (Nanopore, cat. no. SQK-LSK114). Next, samples were loaded on a PromethION Flow Cell (Nanopore, cat. no. FLO-PRO114M) sequenced with the PromethION P2 Solo device (Nanopore, cat. no. PRO-SEQ002) for 48 h, reaching more than 100 million reads.

#### Data analysis

Raw Nanopore signals were basecalled using Dorado (v0.7.3, https://github.com/nanoporetech/dorado) with the dna_r10.4.1_e8.2_400bps_sup@v5.0.0 model. Reads shorter than 200 bp or longer than 10 000 bp or with a mean quality score below 10 were filtered out. Demultiplexing based on single-end barcodes was performed using the nanoMux module of nanoSweet (v1) with up to one mismatch allowed, scanning the first 400 bp at either end of each read, including trimming of reverse adapters and indices (nanoSweet, Zenodo, https://doi.org/10.5281/zenodo.15052178). Forward adapter sequences were removed using Cutadapt (v5.0). Viewpoint-specific demultiplexing was then performed using Cutadapt's linked adapter functionality, retaining only reads matching both viewpoint-specific sequences. For each read, only the first 100 bp downstream of the forward primer—representing interacting regions—were retained. These processed fragments were aligned to the reference genome using Dorado, sorted and indexed using samtools (v1.21) [30], and genomic coordinates were extracted using BEDTools (v2.31.1) [31]. Interacting fragments within 10 kbp of the viewpoint were filtered and the remaining fragments were binned within 250 kbp bins in chr11 down (starting at chr11:69 500,0000)

### Tiled-C data generation and analysis

#### Viewpoint selection and oligo pool preparation

To determine the regions of interest, we evaluated publicly available Hi-C data of GM12878 cells from Rao *et al.* [4]. We selected regions comprising 5 TADs surrounding the breakpoints in chromosomes 11, 14, and 18, and TAD borders were further evaluated using another publicly available dataset [32]. Then, oligo pools targeting DpnII cutting sites within the selected regions (hg38: chr11: 66 940 000 - 71 860 000; chr14: 103 980 000 - 107 043 718; chr18: 61 460 000 - 64 500 000) were designed using the Oligo tool [33] and filtered for repetitive sequences. Double-stranded, biotinylated DNA oligonucleotides were purchased from Twist Biosciences (Twist NGS Target Enrichment Oligonucleotide Panel, cat. No. 100 533), and reconstituted following manufacturer’s instructions to a stock concentration of ≥1 μM for each unique oligonucleotide.

#### Sample preparation

Tiled-C was performed as previously described [34] with minor modifications. Briefly, 5 million cells were collected in single-cell suspensions, fixed in 2% (vol/vol) formaldehyde and incubated for 10 min at RT while tumbling or rotating. Formaldehyde was quenched with 1 M cold glycine, and the cell pellet was lysed in ice-cold lysis buffer (1 M Tris, pH 8; 5 M NaCl; 10% (vol/vol) Igepal CA-630; 1 tablet of cOmplete EDTA-free Protease Inhibitor Cocktail tablets, Roche, cat. no. 04 693 132 001) for 30 min. Cells were pelleted and resuspended again in 1x DpnII buffer (NEB, cat. no. B0543), snap-frozen and stored at −80°C for short-term storage. Digestion with DpnII enzyme (NEB, cat. no. R0543) was performed as described [34], followed by ligation using the NEBNext Quick Ligation kit (NEB, E6065) and DNA extraction with phenol-chloroform-isoamyl alcohol (PCI) and ethanol precipitation. DNA pellets were resuspended in TE buffer and kept at −20°C until library preparation. Quality control of each step was performed in a 15 (wt/vol) agarose gel using 1x Tris acetate-EDTA buffer, run at a moderate speed (70 mA) using 1 μl GeneRuler DNA ladder (Thermo Scientific, cat. no. SM0331), 15 μl of each control and 5 μl of 3C library. Qubit dsDNA High-Sensitivity assay kit (Thermo Scientific, cat. no. Q32854) was used to determine the final DNA concentration, and a standard yield of 60–75% of input DNA was obtained (> 15 μg from 5 million cells). Only libraries with >70% digestion efficiency were taken for the next step.

#### Library preparation and sequencing

Before library preparation, 6.5 μg of DNA for each sample was diluted in 1x TE buffer and sonicated using Covaris to a final mean size of around 250 bp. Around 2 μl of the sample pre- and post-bead clean-up (SPRIselect beads, Beckman Coulter, cat. no. B23317) were run in the TapeStation D1000 as a quality control to check peak size. Then, samples were end-repaired and adaptor-ligated using the NEBNext Ultra DNA Library Prep Kit for Illumina (NEB, cat. no. E7645S), and indexed in duplicate by PCR using the Herculase II Fusion DNA Polymerases kit (Agilent, cat. no. 600 677) and the NEBNext Multiplex Oligos for Illumina, Primers Set 1 and 2 (NEB, cat. no. E7335 and E7500). We proceeded by pulling the samples together, and 2 μg of DNA from each sample were dried out using a Vacuum Speedback machine. Samples were then captured with the probe pools using the fast hybridization mix, fast hybridization enhancers and amplification primers from the Twist Fast Hybridization and Wash kit (Twist, cat. no. 104 180), and the blocker solution and universal blocker from the Twist Universal Blockers kit (Twist, cat. no. 100 578). After streptavidin washes using MyOne Streptavidin C1 Dynabeads (ThermoFisher, cat. no. 65 001), capture amplification was performed by PCR of 22.5 μl per sample in nine cycles, using the KAPA HiFi HotStart ReadyMix kit (Roche, cat. no. KK2602). After AMPure XP beads clean-up, DNA was purified with ethanol and resuspended in PCR-grade water. Final library quantification was performed using high-sensitivity Qubit assay and the KAPA Library Quantification Kit (Roche, cat. no. KK4824) with 1:10 000 and 1:20 000 dilutions. Fragment analyzer to detect the size of the final peaks was also performed. Libraries were diluted to a final concentration of 4 nM and sequenced with the NextSeq 500/550 HighOutput Kit v2.5 (75 cycles, Illumina, cat. no. 20 024 906), with 75 bp reads and pair-end sequencing, obtaining around 1 billion sequenced raw reads (62 million reads per sample in average).

#### Data analysis

Sequencing results were analyzed using the CapCruncher pipeline, as specified by Downes *et al.* [34]. Shortly, sequencing replicates were merged before alignment, and after a quality control test of the fastQ files, sequencing data was aligned to a custom hg38 genome containing an additional chromosome (named chromosome 23) constructed by concatenation of the targeted regions in chromosome 14, 11, and 18, respectively, and excluding alternative haplotypes. The captured regions were consequently masked from the original chromosomes to avoid multiple alignment errors. Following alignment, data was filtered for duplicates and unmapped reads using *pairtools*, and matrices from biological replicates were aggregated and scaled based on the number of restriction sites per fragment. Final read counts were normalized by iterative correction and eigenvector decomposition (ICE) [35] using HiCExplorer (v. 3.7.2) and cooler (v. 0.9.3) packages. Next, breakpoint regions were defined based on the drop in contact frequency from one side of the translocation to the other (Supplementary Table S3). From the constructed chromosome 23, reads from each derivative or chromosomal fragment were selected and re-scaled to be able to compare them among cell lines. Tiled-C heatmaps were visualized using the HiContacts package (v. 1.6.0) in RStudio. From the output data, insulation scores (IS) on strong boundaries were calculated using Cooltools too (v. 0.6.1) and represented in R. TAD borders were called using HiCExplorer (v. 3.7.2). In addition, read counts were used to calculate interaction frequencies of Tiled-C reads from the breakpoint region (chr11:69 677 180–71 777 180 – hg38) toward the entire arm of chromosome 11 down (from chr11:72 000 000 – hg38), using a window size of 250.000 bp and setting the total number of interactions to this segment to 100%.

### 3D FISH data generation

#### Sample preparation

Suspension cells were prepared as recommended by the probes’ manufacturer (Cytocell Ltd., Sysmex Group Company), with modifications to preserve 3D structure. Briefly, 1 million Z-138, JVM-2 and GM12878 cells were cultured in poly-L-lysine pre-coated chambered wells (Nunc^TM^ Lab-Tek^TM^ II, Thermo Fisher, cat. no. 154 526) for 45 min at 37°C. Cell attachment was monitored under the microscope. Then, cells were washed with 0.3x PBS for 30 s to pervent shrinking, and fixed with 4% PFA/1x PBS for 10 min at RT. Fixation was quenched with 0.1 M Tris-HCl pH 7.4 for 10 min at RT, and cells were then permeabilized in a fresh solution of 0.1% saponin/0.1% Triton X-100 in PBS for 10 min. Following two washes with PBS for 5 min, cells were incubated for at least 20 min, but majorily overnight, in 20% glycerol in PBS at 4°C. On the next day, the slides were submerged in liquid nitrogen for 4 cycles of freezing and thawing, and washed with PBS followed by another incubation in 0.1 M HCl for 30 min. Cells were then permeabilized with 0.5% saponin/0.5% Triton X-100 in PBS for 30 min. Next, slides were immersed in 2x SSC for 2 min, and dehydrated successively in 70%, 85%, and 100% ethanol dilutions. Slides were air-dried and 10 μl of pre-warmed fluorescent probe mixtures were spotted onto the slide (see section below for probe details). Fixed cells were then covered with a glass coverslip (24 × 24mm, Menzel-Gläser, VWR, cat. no. 630–2104) and sealed with rubber solution glue (Marabu GmbH & CO, cat. no. 2901 10 000). DNA and probes were denaturalized together on a hotplate for 2–5 min at 78°C, with a weight on the slide, and covered from the light. Slides were then incubated overnight at 37°C in a humid, light-proof chamber inside an incubator. On the following day, coverslips and all traces of glue were removed, and slides were immersed in 0.4x SSC solution for 2 min at 72°C in a water bath. After washing the slides with 2x SSC + 0.05% Tween20 at RT for 30 s, they were incubated with DAPI (0.1 μg/ml) diluted in 2x SSC for 10 min, washed twice with 2x SSC and air-dried. After that, 15 μl of antifade mounting media (Vectashield, Palex Medical SA, cat. no. 416 397) were added, and a coverslip was applied and sealed with nail polish. Slides were directly imaged or stored at −20°C.

#### Probes

Break-apart probes for the *CCND1* and IGH loci were purchased from Cytocell (Sysmex Company Group, Oxford Gene Technology, cat. no. LPS 030 and LPH 014, respectively), which target upstream and downstream of the gene of interest with two fluorophores (Supplementary Table S4). For each experiment, 10 μl of the corresponding break-apart probes were mixed and pre-warmed for 5 min at 37°C. Then, the probe mixture was spotted onto the cell layer on each slide, and the following steps were performed as stated in the previous section.

#### Data acquisition

High-throughput imaging was performed using a fully motorized inverted Zeiss linescan confocal LSM980 unit with Airyscan 2 system, controlled by Zen Blue 3.2 software. Images were acquired using a Plan-Apochromat 63x/1.4 oil immersion objective and Zeiss 1.518 refractive index (Abbe number: 45) oil immersion media. For excitation, 405 nm (for DAPI), 488 nm (Alexa Fluor 488) and 561 nm (Texas Red) laser diodes were used. The emitted light was detected in a sequential mode with the emission filters: 465 (DAPI), 517 (Alexa Fluor 488) and 592 (Texas Red) with an Airyscan detector. Images covering a 20 μm axial range completely covering the cell nuclei volume were acquired, with stacks of 0.13 μm and a voxel size of 73 × 73 × 130 nm^3^. Zoom factor was 3.6, scanning was unidirectional and line average was set to 2, with an image size of 35.7 × 35.7 μm^2^. Imaging was conducted in a controlled environment to maintain sample integrity, and exposure time that was kept for all samples. Before acquiring images, 200 nm multi-color beads (Invitrogen™ TetraSpeck fluorescent microspheres, cat. no. T14792) were imaged with exactly the same settings and in the same environmental conditions, to correct for chromatic shift in the biological sample.

### 3D FISH analysis

#### Chromatic aberration correction

First, chromatic aberration was corrected closely following the procedure by Kozubek *et al.* [36], using the TetraSpeck beads images which allowed for the detection of chromatic shifts between channels. To create a per-dataset correction, first, dots were identified in each channel of the bead images using a Difference of Gaussians (DoG) filter; second, dots were localized to sub-pixel precision by Maximum Likelihood (ML) fitting using a Gaussian model for the dot shape. Clustering was applied to find corresponding pairs, giving the coordinates of the same bead in two channels at a time. With corresponding pairs as input, a correction transform was found by the least squares method, specifically a polynomial correction of order 2 was used in the lateral plane and of order 0 (a constant shift) in the axial direction, which aligns all channels to DAPI. Then, the per-dataset correction transform was eventually applied to the coordinates of the detected dots, i.e. after the dot detection procedure. After correction, the largest mean square error between the corresponding pairs was found to be 0.25 pixels, indicating that subpixel precision was reached.

#### Nuclei segmentation

The DAPI channel was binarized using the following steps: (1) Otsu’s method was used to create an initial binarization in MATLAB (*imbinarize*, MathWorks, Natick, MA). (2) Then, Iterated Conditional Modes (ICM) were used to improve the initial segmentation [37], and the mean variance of pixels classified as foreground with respect to the background from the previous step was used for initialization. The homogeneity parameter, β, was set to 1.5. (3) Face-connected regions were identified and their holes were filled using morphological operators. (4) Nuclei overlapping the image edges were discarded. (5) Nuclei were split using the watershed cuts (WSC) from Cousty *et al.* [38]. (6) The resulting masks were relabeled again. Finally, G1 cells were selected by excluding those cells in which the total DAPI intensity was outside the expected range (1e9-3.5e9), which was set manually by inspecting the histogram of the per-nuclei DAPI values.

#### Dot detection

Images were convolved with a Laplacian of Gaussian (LoG) filter with *σ = [1.55,1.55,3.29]*, considering [σ_x_,σ_y_,σ_z_]. The image was then partitioned into patches by the WSC algorithm as explained previously [38]. For each patch, *P*, two features were used, *F1 = (max(P) - min(P))* and *F2 = - min(LoG(P))*, to separate the patches representing the true dots from the background. For each remaining patch, the center of mass (COM) was calculated. For this purpose, the min value was subtracted from the region, meaning that only the values higher than the min value of the local patch were used for the COM calculation. To avoid over-segmentation, known to possibly occur for probes covering large genomic regions, patches closer than *d_0_ = 12* pixels were merged.

#### Quality control

A final quality control was performed, and only those nuclei that met all criteria for each nucleus were kept for further analysis. First, each nucleus needs to have two pairs of dots, corresponding to the upstream and downstream segments of *CCND1*/IGH loci in the two alleles of a diploid cell line. Second, each cell needs to have two wild-type copies (GM12878) or a wild-type and a translocated copy (Z-138 and JVM-2). Due to the nature of the break-apart probes, dots will localize close together on a wild-type chromosome, while being further away when hybridizing to translocated chromosomes. Given that each of our cells carries at least one wild-type allele, we assigned the dot pair with minimum distance first, while the remaining dots formed the second pair. To properly assess whether the assigned dot pairs represent a wild-type (non-broken) or translocated (broken) chromosome, we built a model for the expected distance in the known wild-type case of GM12878 cells. Therefore, we estimated the chi-square-3 probability of a dot pair being wild-type using its square Euclidean distance, normalized for the σ_x_, σ_y_, and σ_z_ values estimated from GM12878. We took a threshold of 0.95 to reject the null hypothesis that pairs come from the non-broken wild-type chromosome, and using this threshold, we classified each dot pair as wild-type or translocated for all cells (two dot pairs per cell). Wild-type nuclei are required to have two non-broken dot pairs, while cells with translocations are required to have one non-broken and one broken dot pair.

#### Measurements

For each nucleus, we measured the distances between each dot and the closest region of the nuclear lamina (inferred from the DAPI signal). The Euclidean distance transform was applied as previously described [39], taking the anisotropic pixel size into account. The distance values were scaled to have a maximum value of 1, meaning that all distance values are relative, where 0 means that the detected dot is located at the nuclear lamina, and 1 means located at the nuclear center. Finally, the distance maps were interpolated over the fitted coordinates of the detected FISH probes, and downstream analyses were performed in RStudio (version 2024.04.0 + 735).

### Chrom3D modeling

#### Deconvolution of Hi-C matrices

To feed the modeling software with diploid models, the Hi-C matrices were deconvoluted as explained next. For the normal B cell and CLL merged (1 Mb resolution) Hi-C matrices, intrachromosomal reads were divided by two and assigned to what we call alleles a and b. Interchromosomal reads were divided by four and assigned to the four possible combinations of alleles (a-a, b-b, a-b, b-a). In MCL, for chromosomes 1–10, 12–13 and 15–22 the same approach was applied as described above. For chromosomes 11 and 14 in MCL, we assigned the same amount of reads to the single wild-type chromosome as observed in normal B cells. The remaining reads were assigned to the derivative alleles. Interchromosomal reads between chromosomes 11, 14, derivative 11 and derivative 11 were set to NA.

#### Chrom3D modeling

Chrom3D [40] was applied to build 100 nuclear models of normal B cells, CLL and MCL. As input the deconvoluted matrices were used, as well as A and B compartment assignments, where B compartments were used as a constraint reflecting peripheral placement at the nuclear lamina. Chrom3D was executed using default settings, with the exception that for each model we shuffled the order in which the chromosomes were fed into the model. Next, per model the nuclear positioning of each chromosome was calculated as the Euclidean distance of its center of mass with respect to the center of the nucleus. In addition, for chromosomes 11 and 14, for each model we calculated the pairwise Euclidean distance per 1 Mb bin.

#### ChimeraX visualization

For visualization of the *in silico*-generated nuclear models, we used ChimeraX (version 1.6). CMM output files of Chrom3D were imported into ChimeraX for whole nuclei visualization. For specific chromosome visualization, the R,G,B values of the CMM files were modified and the files were filtered to display selected beads with desired colors. The modified files were then re-imported into ChimeraX.

### CRISPR-Cas9 genome editing to generate translocations

#### sgRNA design

Single guide RNA molecules were designed to target the region where translocations usually cluster in MCL patients and synthesized by Synthego (Redwood City, CA, USA). The targeted regions of interest in hg38 are chr11:69 531 666–69 531 685 (sequence: GAACCCAGGGTCCATTCCAC) and chr14:105 863 775–105 863 794 (sequence: TCCCTAAGTGGACTCAGAGA).

#### CRISPR-Cas9-based genome editing

Genome editing of the healthy cell line GM12878 to generate translocation t(11;14) *de novo* was conducted using the Neon^TM^ electroporation system. Briefly, RNP complexes were formed by combining 3 μM of each sgRNA and 2 μM SpCas9 NLS recombinant nuclease in R buffer (Neon^TM^ NxT Eletroctroporation System 10-μL Kit, cat. no. N1025, ThermoFisher) for 10–20 min at RT. After RNP complex formation, 0.3 million cells were electroporated using the Neon^TM^ device (Neon^TM^ NxT Electroporation System, NEON1S, ThermoFisher) with 1 600 V, 20 ms, and 1 pulse. Cells were immediately transferred to pre-warmed 12-well plates with 1 ml of RPMI-1640 media supplemented with 20% FBS without antibiotics and kept at culture conditions until further analyses were performed.

#### Digital PCR analysis

The presence and quantification of the t(11;14) translocation were assessed using the Digital LightCycler Analyzer System from Roche (Roche, cat. no. 09 274 804 001). Briefly, primers targeting both sides of the breakpoint region were designed, (forward: CGATCTTGCAGTCCTACA; and reverse: TGATGGAGTAACTGAGCC), as well as a probe that hybridizes upstream of the translocation breakpoint in chromosome 14 (AGACACATTCCTCAGCCATCACT / 56-FAM / AGACACATT / ZEN / CCTCAGCCATCACT / 3IABkFQ). Hence, only when the translocation is present, a PCR product is generated, and the fluorophore tag will emit light. Based on a control region targeting chromosome 14 upstream of the breakpoint (forward primer: GAAGCGGAGAGAGGTCAC; reverse primer: TGGCCTTTGCAGCTAATA; and probe sequence: CCAAGTCCGGCCACAGATGTC / 5SUN / CCAAGTCCG / ZEN / GCCACAGATGTC / 3IABkFQ), the fraction of translocated over total alleles was calculated and converted to the fraction of cells carrying translocations (considering that the vast majority of the cells are heterozygous for the translocation). Primers and probes for the digital PCR were purchased from IDT and diluted to a stock concentration of 100 μm upon reception. The dPCR kit and plates used were purchased from Roche (Digital LightCycler 5x DNA Master, cat. no. 09 393 544 001; Digital LC Uni Plate, cat. no. 09 033 696 001).

### Bulk RNA-seq data generation and analysis

#### RNA-seq data of patient data

RNA-seq data from 5 patients and 12 control samples were mined from a previous study [24]. We used the raw and FPKM estimates for downstream analysis.

#### RNA-seq data of genomically engineered cells carrying translocations

Single-stranded RNA-seq data was generated as previously described [41]. Briefly, RNA was extracted and purified using the RNeasy kit for RNA purification (Qiagen, cat. no. 74 104), from three biological replicates of GM12878 cell populations containing around 10% of cells with the translocation t(11;14), and six GM12878 control samples. Controls 1–3 were electroporated with RNPs containing single guide RNAs (sgRNAs) targeting chromosome 11, while controls 4–6 contained sgRNAs targeting chromosome 14 and Cas9. RNA was purified using the RNeasy kit for RNA purification (Qiagen, cat. no. 74 104) and retro-transcribed into cDNA. Then, a qPCR of CCND1 was conducted (primer sequences: CCTGTCCTACTACCGCCTCA and TGGGGTCCATGTTCTGCT) using the Lightcycler 480 (Roche) with SYBR green mix (Roche, cat. no. 04 707 516 001). Next, libraries were prepared for next-generation sequencing by the genomics facility (CRG) and adapter-ligated libraries were amplified and sequenced using 100 bp paired-end reads in a Next-Seq (Illumina). The quality of the raw data was checked using FastQC (v. 0.11.5), and reads were trimmed for low quality and adapter using skewer (v. 0.2.2). The percentage of reads mapping to ribosomal RNA was assessed using riboPicker (v. 0.4.3), and trimmer reads from the 12 samples were mapped to the human Gencode release 46 transcriptome using Salmon (v. 1.5.1). Transcript level estimates were summarized using the R package *tximport* (v. 1.32.0), and raw as well as transcripts-per-million (TPM) counts were used for downstream analysis.

#### Differential gene expression analysis

Differentially expressed genes were defined using the DESeq2 R package (v. 1.44.0) [42]. Briefly, only protein-coding genes expressed in at least one sample (TPM or FPKM values > 1) were included for downstream analysis. Next, the *DESeq* function was used to normalize, estimate dispersion, model fitting and hypothesis testing. For primary samples, the 5 MCL samples were grouped and compared to the 12 healthy mature B-cell samples, merging samples from naive, germinal center, memory and plasma B cells. Only those protein-coding genes with a log2 fold change > 1 in MCL samples compared to all healthy samples and an FDR value < 0.1, while being expressed in at least 3 out of the 5 MCL samples, were considered as overexpressed. Downregulated genes were assigned having a log2FC < −1 and an FDR < 0.1. The genetically engineered cells (Tx1-Tx3) were compared to C1-C6 controls treated with only one sgRNA to exclude the effect of double-strand breaks on chromosomes 11 and 14, and other possible off-target effects. Of note, considering that only 10–12% of the cells are translocated, a 2-fold overexpression in cells with translocations would result in a global fold change of 1.1. Therefore, differentially expressed genes were defined as upregulated when having a fold change > 1.1 and an FDR < 0.1 in Tx1-Tx3 compared to C1-C6. PCAs were generated using the *prcomp* function from the state R package (v. 3.5.1), using vst-transformed data.

#### Gene ontology analysis

Gene ontology analyses were performed using the R package EnrichGO from clusterProfiler (v. 4.12.3) [43], and corroborated using the online enrichR tool [44, 45]. All protein-coding genes in the human transcriptome were taken as the background list when compared to global upregulated genes. On specific chromosomal regions (such as chr11 up, chr11 down, and chr14 up), only protein-coding genes on the same regions were taken as background.

#### Permutation tests

From all protein-coding genes within the investigated chromosomal segment (chr14 up, chr11 up, and chr11 down) a random set of genes was picked, equal in number to the set of upregulated genes. For each 3 Mb bin, the number of random genes was compared to the number of true upregulated genes in that window. This was repeated 10 000 times and *P*-values were calculated as the frequency in which the number of random genes was higher than the number of upregulated genes plus one, overall divided by 10 000.

### Singleron single-cell RNA-seq data generation and analysis

#### Cell isolation and library preparation

For scRNA-seq analysis, we generated single-cell suspensions of genetically engineered GM12878 cells (samples Tx1-Tx3). Cell partitioning, lysis, barcoding and library generation processes were performed by the IRB genomics facility using the GEXSCOPE Single Cell RNA Library kit (HD) from Singleron (cat. no. 4 180 031) and following manufacturer’s protocol. Libraries were quantified using Qubit HS and sequenced on an Illumina NovaSeq 6000 S2 platform with an average depth of approximately 20k reads per cell.

#### Data preprocessing

Raw sequencing reads were processed using the CeleScope (v. 2.0.7 pipeline. Briefly, reads were mapped to the GRCh38 reference genome using the STAR aligner within CeleScope, and gene-level expression matrices were generated. Subsequent analyses were performed using R (v. 4.2.1) and the Seurat package (v. 5.0.1). Cells with high mitochondrial RNA content (>20%), low gene count (<500 or > 6 000 genes detected), or low/high read count (<1000 or > 30 000 reads) were filtered out to remove low-quality or dying cells. Doublets were also identified and removed using scDlbFinder (v. 1.12.0).

#### Data normalization and clustering

Single-cell transcriptomes of the three replicates were combined and log-normalized, and the top 3000 variably expressed genes were identified. Immunoglobulin, HLA, mitochondrial, and ribosomal genes were filtered out, and cell cycle phase scores were calculated using the *CellCycleScoring* function from Seurat. Cell cycle phase scores, read counts and percentage of mitochondrial reads were regressed out during data pre-processing to correct for sources of unwanted variation. PCA analysis was performed using the same package. Significant components were selected, and cells were clustered using the Louvain algorithm. Cells were classified based on CCND1 expression (at least one transcript per cell) and visualized using UMAP (performed on the first 30 PCs). Differential genes between CCND1-positive and -negative cells were assigned by the FindMarkers function in Seurat using an FDR threshold of 0.1.

#### Active promoter identification

For each gene, chromatin states in GM12878 were assessed within 1500 bp up- and downstream of the TSS using previously published data [32]. When active promoter or promoter-associated enhancer marks were present, the promoter was considered active.

### SMART-seq2 single-cell RNA-seq data generation and analysis

#### Cell isolation and library preparation

Full-length single-cell RNA-seq libraries were prepared using the SMART-seq2 protocol [46] with minor modifications. Briefly, freshly harvested single cells of genetically engineered GM12878 cells were sorted into 96-well plates (four plates each for samples Tx1 and Tx2, two plates for sample Tx3) containing 2 μl of lysis buffer (0.2% Triton X-100, 1 U/μl RNase inhibitor, Thermo Fisher Scientific, cat. no. 10 777 019). Reverse transcription was performed using SuperScript II (Thermo Fisher Scientific, cat. no. 18 064 014) in the presence of 1 μM oligo-dT30VN (IDT), 1 μM Template-switching oligonucleotides (QIAGEN), and 1 M betaine. cDNA was amplified using the KAPA Hifi Hotstart ReadyMix (Kapa Biosystems, cat. no. 09 420 398 001) and IS PCR primer (IDT), with 22 cycles of amplification. Following purification with Agencourt Ampure XP beads (Beckman Coulter, cat. no. A63881), product size distribution and quantity were assessed on a Bioanalyzer using a High Sensitivity DNA Kit (Agilent Technologies, cat. no. 5067–4626). A total of 140 pg of the amplified cDNA was fragmented using Nextera XT (Illumina) and amplified with with double indexed Nextera PCR primers (IDT). Products of each well of the 96-well plate were pooled and purified twice with Agencourt Ampure XP beads (Beckman Coulter, cat. no. A63881). Final libraries were quantified and checked for fragment size distribution using a Bioanalyzer High Sensitivity DNA Kit (Agilent Technologies, cat. no. 5067–4626). Libraries were sequenced on the NextSeq500 platform with an average depth of approximately 0.5 M reads per cell.

#### Data preprocessing

SMART-seq2 data was analyzed using the allele_specific_RNAseq pipeline [47] (https://github.com/biocorecrg/allele_specific_RNAseq) with the human genome assembly GRCh38 and the annotation version 112 from Ensembl as references. To avoid alignment bias, the reference genome was masked for known genetic variants (file: 250 307_chr11.alleseq.GIAB.overlap.GM12878.vcf, see supplementary material). This file contains phased heterozygous SNP data for GM12878 mined from two sources, the Genome in a Bottle Consortium (GIAB) and Rozowsky *et al.* [48] The concordant phased heterozygous SNPs from these two sources were selected for downstream analysis. Briefly, the allele_specific_RNAseq pipeline assesses read quality with FastQC (https://www.bioinformatics.babraham.ac.uk/projects/fastqc/) followed by aligning reads using STAR [49], with the WASP filtering algorithm [50] enabled to correct for mapping biases and assign each read to a given allele. As strand-specific information was not preserved during library preparation, alignment was performed without assuming RNA strandness. A custom Python script based on pysam efficiently separates the alignment generated by STAR into four different files: Allele A, Allele B, neither A nor B, and ambiguous (both variants found). Cells with low mapped reads count (<50 000) were filtered out to remove low-quality cells.

#### Allele-specific analyses

Cells without gene counts in CCND1 were considered not to carry the translocation (*n* = 701), and haplotypes A and B were assigned to the maternal or paternal alelle based on the biased representation of SNPs in known paternally- (KCNQ1OT1) and maternally- (KCNQ1 and ZNF215) expressed transcripts in these cells. Next, cells with at least five allele-specific reads in CCND1 assigned to one haplotype, and no reads to the other haplotype, were categorized to contain the t(11;14) toward the maternal (*n* = 50) or paternal (*n* = 27) allele of chromosome 11. Finally, for 29/50 upregulated genes in our Singleron scRNA-seq data (excluding CCND1), i.e. those containing at least 10 allele-specific reads in the control cells and in at least one pool of cells harboring translocations, the percentage of maternal and paternal transcripts were assigned using pseudo-bulk counts.

## Results

### MCL cells undergo restructuring of the interchromosomal interaction landscape

**Figure 1.**
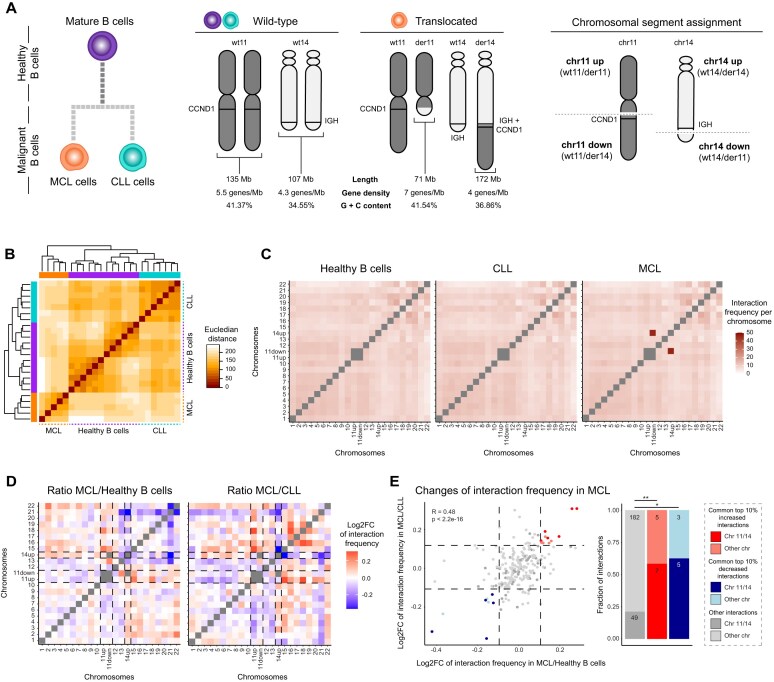
The interchromosomal 3D landscape in MCL. (**A**) MCL and CLL arise from healthy mature B cells upon malignant transformation (left), with the translocation t(11;14) in MCL resulting in changes in chromosomal characteristics such as length, gene density, and GC content (middle). Chromosomal segments of chromosomes 11 and 14 are assigned as “up” and “down” based on being located up- or downstream of the translocation breakpoints marked as dotted line (right). (**B**) Dissimilarity matrix comparing the Hi-C matrices of all interchromosomal reads among samples. (**C**) Heatmap of interchromosomal interaction frequencies between chromosomes after ICE-normalization. (**D**) Log2 fold-change of the ratio of interaction frequencies between MCL and healthy B cells (left) and MCL and CLL (right). Dashed lines highlight translocated chromosomal segments. (**E**) Log2 fold-change of interchromosomal interaction frequencies in MCL versus healthy B cells (*x*-axis) or CLL (*y*-axis) with the top 10% common increased/decreased interaction frequencies shown in the upper-right or lower-left quadrants, respectively (left). Barplots show fractions of increased, decreased and non-changed interactions in chromosomes 11 and 14 compared to other chromosomes (right). Numbers in the bar plots represent the total number of pairwise interactions in each bar. der = derivative, wt = wild-type, down = downstream of the breakpoint position, up = upstream of the breakpoint position, **P-value < 0.01, *P-value < 0.05.

MCL cells are malignant B cells that frequently carry a translocation involving the balanced exchange of DNA between chromosomes 11 and 14, leading to the juxtaposition of the *CCND1* proto-oncogene with the IGH enhancer. While the effects of the resulting CCND1 overexpression are widely reported, other possible consequences of the translocation remain unclear. For instance, the transfer of chromosomal material leads to the formation of two new derivative chromosomes, with altered length, gene density and GC content (Fig. 1A). These variables have previously been associated with the spatial positioning of chromosomes within the cell nucleus [8, 51–53], suggesting that translocations possibly affect CT positioning.

To understand the effect of the MCL-related SV t(11;14) on nuclear architecture, we first analyzed publicly available *in situ* Hi-C data of five MCL patients at diagnosis, and compared their interchromosomal interaction frequency landscape to mature B cells from healthy individuals as negative controls [24] (Supplementary Table S1). The 12 control samples comprised naive, germinal center and memory B cells as well as plasma cells. Additionally, we compared the alterations in MCL to seven chronic lymphocytic leukemia (CLL) samples—mature B-cell tumors without translocations—to differentiate the effects of malignant transformation from those associated with the translocation *per se* (Fig. 1A). Analysis of the interchromosomal interactions at the megabase (Mb) scale—either including (Fig. 1B) or excluding (Supplementary Fig. S1A) the interactions between translocation partners—revealed that MCL samples cluster separately from healthy B cells and CLL. Next, we grouped interchromosomal interactions per sample type, masking donor-specific effects (Supplementary Table S5) and leaving only common effects in play. Indeed, chromosome-wise comparisons showed a high, MCL-specific interaction frequency between the lower part of chromosome 11 (chr11 down, downstream of the translocation breakpoint) and the upper part of chromosome 14 (chr14 up, upstream of the translocation breakpoint), confirming the presence of the t(11;14) translocation as the only common SV (Fig. 1C). Of note, the two Mb bins downstream of the translocation breakpoint on chromosome 14 (chr14 down)—located on der11 upon the translocation—showed low numbers of 3D interactions likely due to poor mappability, and were therefore removed from the analysis.

Next, after removing the intrachromosomal interactions between translocation partners, we calculated the pairwise chromosomal interaction changes among MCL, healthy B cells and CLL (Fig. 1D, Supplementary Fig. S1B). Our results unveiled consistent MCL-specific interchromosomal interaction alterations involving chromosomes 11 and 14. Particularly, within the common top 10% of alterations in MCL compared to healthy B cells and CLL, interactions involving chromosomes 11 and 14 were significantly enriched with respect to the remaining interactions (2.75-fold for commonly increased, and 2.95-fold for commonly decreased interactions, with respective chi-squared p-values 0.0086 and 0.0206) (Fig. 1E). Interactions from the upper part of chromosome 11 (chr11 up, upstream of the translocation breakpoint) particularly increased toward short chromosomes, mainly chromosomes 15 and 18, while they decreased to long chromosomes, such as chromosomes 6 and 7. In contrast, increased interactions from chr14 up mainly occurred toward long chromosomes, such as chromosomes 3 and 7, while decreasing interactions to short chromosomes like chromosomes 15, 20, and 21 (Supplementary Table S6). From these results, we conclude that MCL cells display global alterations of their interchromosomal 3D interaction landscape, mainly associated with the chromosomes that are directly affected by the translocation.

### Derivative 11 shifts toward the nuclear center in MCL cells

**Figure 2.**
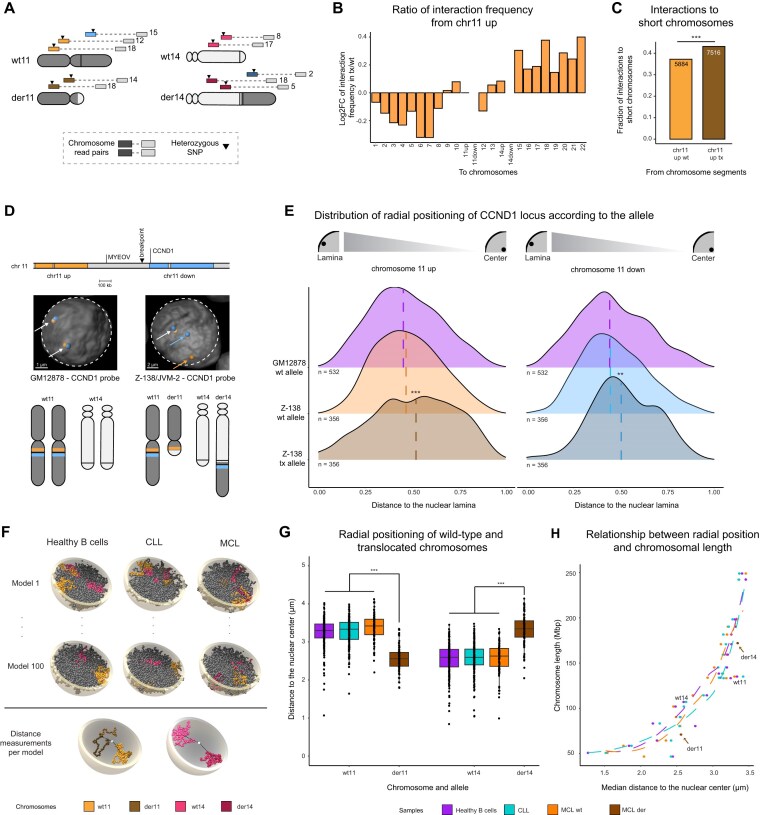
Allele-specific analysis of translocated chromosomes in MCL. (**A**) Classification of Hi-C reads to the wild-type or translocated allele in Z-138. Hypothetical chromosome numbers are indicated, linked via the dashed line. (**B**) Log2FC of the ratio of Hi-C interactions between translocated and wild-type chr11 up toward all other chromosomes in Z-138. (**C**) Fraction of Hi-C reads from chr11 up (wild-type or translocated) to short chromosomes (chromosomes 13–22) in Z-138. Numbers in the bar plots represent the total number of interactions in the given bar. (**D**) Position of the *CCND1* breakapart probe in wild-type and translocated chromosome 11. Two example images were cropped from the original files. Dashed lines delimit the nuclear lamina, while arrows point to wild-type or translocated segments of chromosome 11. (**E**) Density plots of the relative distribution of imaging-based radial position of chromosome 11 in Z-138 compared to control (GM12878), 0.0 indicates the closest pixel to the lamina and 1.0 the most central position. Dashed lines represent the median. (**F**) Graphical representation of two (out of 100) randomly chosen models, colored parts represent chromosomes 11 and 14 (wild-type and translocated). Dashed lines outline the measurements from the central mass of each chromosome to the nuclear center (lower panel). (**G**) Boxplots with the *in silico* modeling-based position of wild-type or translocated chromosomes 11 and 14. Healthy B cells and CLL contain 200 measurements for each chromosome, while in MCL the two alleles are divided into 100 wild-type and 100 derivative alleles. (**H**) Relationship between the *in silico* modeling-based radial position and chromosome length. The length of derivatives 11 and 14 was calculated according to the breakpoint bin in MCL patients. Dashed lines indicate the estimated loess curve for each sample, the curve in MCL was calculated without considering the derivatives indicated by the arrows. der = derivative, wt = wild-type, tx = translocated, down = downstream of the breakpoint position, up = upstream of the breakpoint position, **P-value < 0.01, ***P-value < 0.001, ns = not significant.

In diploid cells, Hi-C analyses elucidate mean interaction frequencies of homologous chromosomes. Hence, allele-specific analyses are crucial to discern whether the MCL-specific interchromosomal interaction differences that we detected for chromosomes 11 and 14 originate from the translocated allele or its wild-type counterpart. To obtain these insights, we performed Hi-C in the MCL line Z-138, which carries the t(11;14) translocation, and simultaneously established bivariate flow cell karyotyping to isolate DNA from its derivative 11, wild-type 11 and wild-type 14 chromosomes. The difference in length and GC content of the derivative 11 compared to the wild-type chromosome (Fig. 1A) leads to the formation of an aberrant chromosome cloud in the bivariate flow cell karyotyping, which was not detected in the control cell line, and could therefore be isolated (Supplementary Fig. S2A). Unfortunately, we could not detect the cloud belonging to derivative 14, possibly because its length and GC content are similar to other chromosomes, avoiding a clear separation. Consequent sequencing of the isolated chromosomes combined with heterozygous SNP assignment, aided by previously published SNP array data [28], allowed us to genotype the derivative and wild-type alleles in an allele-specific manner, enabling the classification of Hi-C reads belonging to the wild-type or the derivative alleles in Z-138 (Fig. 2A, Supplementary Fig. S2B, Supplementary Tables S2 and S7).

The clear gain of interactions between the translocated chromosomes 11 and 14 in comparison to the wild-type chromosomes confirmed the correct classification of Hi-C reads (Supplementary Fig. S2C). We furthermore detected an increased number of reads between the considered wild-type chromosome 14 and chromosome 8, which appeared to represent a t(8;14)(q24;q32) translocation that was previously reported [54] (Supplementary Fig. S2C). From this result, we concluded that Z-138 does not carry a wild-type copy of chromosome 14 to compare with derivative 14. Therefore, chromosome 14 was excluded from further analysis in Z-138. After removing the translocation-based intrachromosomal read counts, we observed a significant increase of interactions from the translocated upper part of chromosome 11 to short chromosomes (13-22) in comparison to wild-type (chi-square test, p-value < 2.2e-16) (Fig. 2B and C), indicating a possible shift of derivative 11 toward the nuclear center. Unfortunately, the low number of interchromosomal read counts that could be assigned to chr11 down (Supplementary Table S7) refrained us from interpreting the allele-specific Hi-C data in Z-138 for this chromosomal segment.

3C techniques cannot assess the physical position of (wild-type and translocated) chromosomes 11 and 14 within the nucleus, as they only define the relative interaction frequency between genomic fragments. For this reason, we performed allele-specific 3D FISH in Z-138 cells, using break-apart probes that specifically bind up- or downstream of the translocation breakpoint (Fig. 2D). This approach enabled us to differentiate the wild-type chromosome 11 from its translocated counterpart, and to compare their radial position within the nucleus (Fig. 2E). The lymphoblastoid cell line GM12878 was used as control, while JVM-2 cells—also carrying the t(11;14) translocation—were included to account for possible variability between MCL cell lines. For each individual imaged cell, probe pairs located up- and downstream of the breakpoint were assigned to the wild-type allele if located close in 3D space (Fig. 2D), falling within the 95% confidence interval established using a model fitted in GM12878 control cells. The remaining, more distant pairs were classified as translocated alleles. Only those cells containing one wild-type and one translocated pair (Z-138 and JVM-2) or two wild-type pairs (GM12878) were considered for further analysis. Next, the 3D distance from each probe to the nuclear surface was measured. In line with our previous Hi-C results, our 3D FISH analyses showed a significant shift of the translocated chr11 up in Z-138, which was located closer to the nuclear center compared to wild-type chr11 up (Fig. 2E, left, Wilcoxon test *P*-value = 2.292e-05). A similar trend was observed in JVM-2 cells (Supplementary Fig. S2D). Similarly, the translocated chr11 down displayed a significant shift toward the nuclear center in Z-138 compared to wild-type chr11 down (Fig. 2E, right, Wilcoxon test *P*-value = 0.01267), although this movement was not detected in JVM-2 (Supplementary Fig. S2D). Additionally, our data points toward a bimodal trend of radial position of the translocated chr11 down segment in both Z-138 and JVM-2 cells, suggesting the possible presence of distinct subpopulations regarding its nuclear positioning. Finally, we also analyzed chromosome 14 positioning in JVM-2 cells, but the data was too sparse—due to a high level of tetraploidy in this cell line—to obtain significant results (Supplementary Fig. S2D).

To further explore the chromosomal radial distribution in the presence or absence of the t(11;14) translocation, we leveraged the patient-derived Hi-C data and generated 100 *in silico* 3D genome models per sample type—MCL, CLL and healthy B cells—using Chrom3D [40]. To obtain diploid cellular models we deconvoluted the Hi-C data considering that, based on our previous results, wild-type chromosomes 11 and 14 in MCL retain the interaction frequencies of normal B cells, while the translocated alleles account for the differences observed between MCL and normal B cells. Each model represents a possible diploid nuclear chromatin conformation structure, inferred by Chrom3D using the interaction frequencies among chromosomes in the Hi-C data (Fig. 2F). Next, we measured the distance from the central mass of each chromosome to the geometric nuclear center, and compared their radial distribution among MCL, CLL and healthy mature B cells. While we did not observe major differences between the wild-type chromosomes in MCL versus CLL and healthy B cells (Supplementary Fig. S2E), we detected a significant shift in position of the translocation partners compared to wild-type chromosomes 11 and 14 (Student's T-test, *P*-values < 2.22e-16) (Fig. 2G). More specifically, we noticed a median inward movement of derivative 11 in MCL over 17.2% of the radial distance (0.86/5 μm, Fig. 2g), displaying a similar nuclear location as wild-type chromosome 14. On the other hand, derivative 14 shifted outwards over a distance covering 14.2% of the radial length (0.71/5 μm), taking a radial position similar to wild-type chromosome 11. Importantly, the indicated shifts mainly target the translocated segments on chr 11 up and 14 up (Supplementary Fig. S2F). These results thus pinpoint a possible exchange of the radial position of these segments within the nucleus, which aligns with their expected position based on the new length of the derivatives on which they reside (Fig. 2H).

Altogether, the combination of our allele-specific Hi-C, imaging and modeling analyses indicates a shift of derivative 11 toward the nuclear center compared to wild-type chromosome 11, strengthening our observations of the MCL patient Hi-C analysis (Fig. 1C). Furthermore, our modeling data suggests a shift of derivative 14 toward the nuclear periphery compared to wild-type chromosome 14, although we do not have sufficient imaging data, nor allele-specific Hi-C data to prove this finding. In addition, the findings regarding the translocated segment of chr11 down, also residing on derivative 14, show contradictory results, moving clearly inwards in our Z-138 3D FISH experiments, and slightly outwards in our 3D modeling data. Therefore, we refrain from drawing conclusions regarding derivative 14 as we consider that further studies are needed to confirm its nuclear positioning.

### Derivative 14 displays ultra-long-range interactions with increased regulatory potential

**Figure 3.**
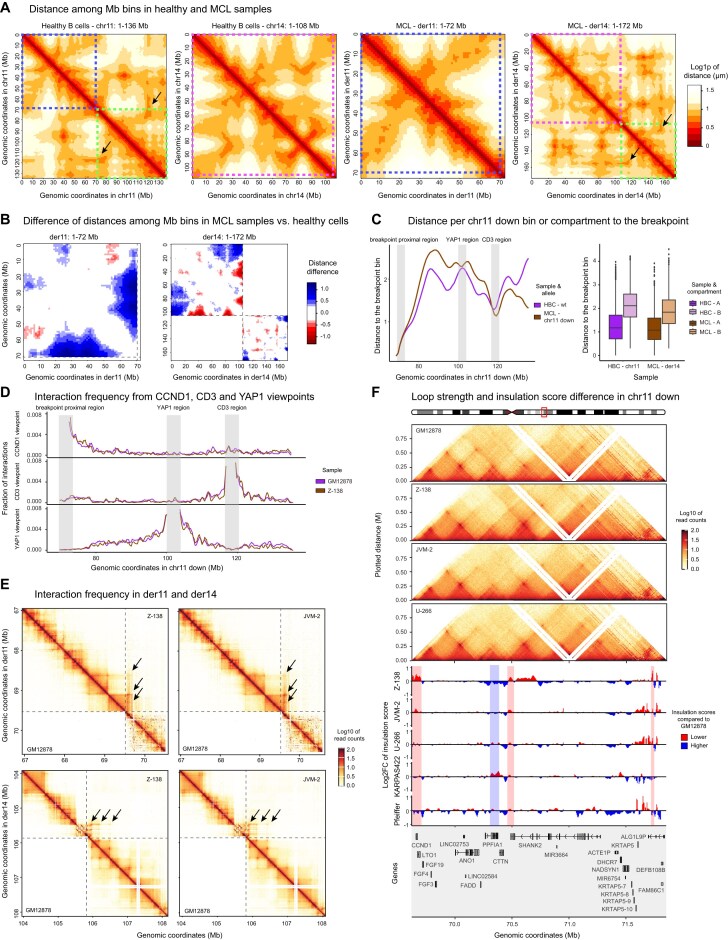
Intrachromosomal interaction landscape of translocated chromosomes in MCL. (**A**) Pairwise log1p-transformed distance between Mb bins on chromosomes 11 and 14 (wild-type, healthy B cells, left panels) and derivatives 11 and 14 (MCL, right panels). Dashed boxes indicate chromosomal segments up- and downstream of the breakpoint. Distances were inferred from the chrom3D modeling output. Arrows indicate the ultra-long interchromosomal interaction in chr11 down. (**B**) Difference between distance of all Mb bin pairs in MCL compared to healthy B cells. Positive values indicate bins located further away from each other in MCL compared to healthy B cells, while negative values indicate bins that get closer together. Dashed lines indicate the translocation breakpoint. (**C**) Distance from each Mb bin (left) or compartment (right) to the translocation breakpoint (Mb bin 69–70). Vertical bars indicate the breakpoint proximal (chr11: 69 500 000 – 73 500 000), YAP1 (chr11: 100 000 000 – 104 000 000), and CD3 (chr11: 116 500 000 – 120 500 000) region. (**D**) Interaction frequencies from different viewpoints to chr11 down measured by 4C-seq. Vertical bars indicate the same regions as in 3C. (**E**) Tiled-C read count heatmaps in derivatives 11 and 14 in MCL cell lines Z-138/JVM-2 (right-upper triangle), and the GM12878 control cell line (left-bottom triangle). Arrows highlight interactions spanning over the breakpoint, indicated by the dashed line. (**F**) Differences in insulation score on chr11 down compared to GM12878. In KARPAS422 and Pfeiffer, chr11 down is not affected serving as wild-type control. Scores below zero indicate increased, while scores above zero indicate decreased insulation scores. Heatmaps represent Tiled-C read counts in the depicted segments. der = derivative, wt = wild-type, down = downstream of the breakpoint, MCL = mantle cell lymphoma, HBC = healthy B cells.

Our previous observations indicate that derivative chromosomes formed upon the generation of the t(11;14) translocation in MCL cells exhibit the most prominent interchromosomal structural alterations compared to wild-type cells. We next investigated whether these effects extend to intrachromosomal interactions. To that end, we first leveraged our MCL, CLL and healthy B-cell *in silico* nuclear models (Fig. 2F) to calculate the distances among all Mb bins on chromosomes 11 and 14 (Fig. 3A). The comparison between wild-type and translocated chromosomal segments revealed a global shift in intrachromosomal distances, predominantly affecting the telomeres and the breakpoint regions on chr11 up and chr14 up in an opposite manner (Fig. 3B). Our data suggests that the gain (chr11 up, Fig. 1A) or loss (chr14 up, Fig. 1A) of linear proximity to the telomere at the breakpoint results in a respectively larger (Fig. 3B, left) or smaller (Fig. 3B, right) 3D distance to the middle segment of the chromosome. We thus hypothesize that telomeric displacement affects global chromosome folding, whereby 3D proximity between telomeres and regions residing in the middle of a chromosome is disfavored. We furthermore observed focal gains of proximity downstream of the breakpoint within derivative 14, suggesting stronger or new long-range interactions upon translocation. To get a more detailed view of this segment, we investigated the 3D distance of all bins on chr11 down to the breakpoint. From this analysis, we observed that healthy B cells and MCL cells share a region on chr11 down, around Mb bins 118–120, that is especially close to the translocation breakpoint in 3D space (Fig. 3C, left). As expected, these interactions mainly involved A compartments (Fig. 3C, right). Chromatin conformation capture analyses by 4C-seq on chr11 down, using the CD3 locus—at Mb 118 involved in the interaction—and the YAP1 locus—at Mb 102 excluded from the interaction—as viewpoints furthermore confirmed the presence of the ultra-long-range interaction. Specifically, the CD3 locus contained between 4.5–8.6 times more interactions to the breakpoint proximal region compared to the YAP1 locus in the investigated B-cell cell lines (Fig. 3D). Interestingly, due to this pre-existing, 50 Mb-spanning ultra-long-range interaction in healthy B cells, the region around Mb bins 118–120 on chr11 down gets close to the strong IGH enhancer in 3D space upon t(11;14) translocation formation. Thus, while translocations do not necessarily alter long-range chromosomal interactions they can exploit pre-existing ones to establish new 3D interactions with potentially strong regulatory impact.

To further understand chromatin conformation changes in regions close to the breakpoint, we generated high-resolution Tiled-C data [33, 34]. We captured large regions (∼3Mb) surrounding breakpoint loci in different cell lines carrying either the t(11;14) translocation (Z-138 and JVM-2) or a cryptic insertion of the IGH enhancer inside chromosome 11 (U-266), and in a wild-type control cell line (GM12878). In order to better characterize the effect of the IGH translocation independently of the translocation partner, we also included two cell lines (KARPAS422 and Pfeiffer) carrying the t(14;18)(q32;q21) translocation, which is commonly found in follicular lymphoma (FL). Principal component analysis (PCA) analysis indicated that cell lines clustered based on their intrachromosomal interaction landscape (Supplementary Fig. S3A). Interestingly, while the 3D genome architecture within the investigated regions on chromosomes 11, 14, and 18 was largely conserved upon translocation formation, *de novo* interactions between the translocation partners could be detected (Fig. 3E, Supplementary Fig. S3B–D).These new interactions reached beyond the neo-TAD formed close to the breakpoint, indicating the formation of long-range interactions spanning a larger region than anticipated. We could also confirm the presence of interactions from the translocation breakpoint in chr11 down—inside the Tiled-C capture region—toward Mb 118–120—outside the capture region—in all cell lines (Supplementary Fig. S3E). Since strong enhancers are associated with high insulated areas, and because we detected stronger focal contacts in the chromosomal segment placed in proximity of the strong IGH enhancer (chr11 down, Fig. 3B right panel), we refined our analysis by comparing insulation scores between cell lines with and without translocations. These analyses showed that derivative 14—consisting of chr14 up, fused to chr11 down (in Z-138 and JVM-2) or chr18 down (in KARPAS422 and Pfeiffer)—displayed many subtle intrachromosomal alterations in insulation scores (Fig. 3F, Supplementary Fig. S3F–H). Both chr11 down and chr18 down for example, which are juxtaposed to the IGH enhancer, alter their loop strength and insulation scores directly downstream of the breakpoint. This effect is stronger upon IGH-translocation formation than upon IGH enhancer insertion (U-266). In addition, just upstream of the breakpoint on chr14 up, we observed clear gains of insulation throughout the segment, except for the part more distal from the breakpoint, which displays stronger interactions within existing loops (Supplementary Fig. S3F and H). The increased insulation score levels are also observed in U-266, in which the IGH enhancer is deleted from one of its copies of chromosome 14 (data not shown). This makes us hypothesize that the observed changes may be due to an IGH enhancer effect, but further research is necessary to better understand this phenomenon. Finally, in agreement with major effects driven by the IGH enhancer, we do not detect any specific alteration in derivatives 11 and 18, which do not associate with the IGH enhancer (data not shown).

Altogether, we show that derivative 14 undergoes a higher level of intrachromosomal chromatin architecture restructuring than derivative 11. Interestingly, this implies that the shift of derivative 11 toward the nuclear center does not drastically change intrachromosomal genome organization. An additional striking finding on chr11 down is the presence of a conserved ultra-long-range interaction spanning the entire chromosome arm in healthy B cells and MCL. The translocation involves the strong immunoglobulin enhancer into this interaction, potentially allowing the spread of its regulatory potential over the entire affected chromosome arm, beyond the previously reported CCND1 overexpression. Therefore, shedding light on gene expression dysregulation in the genomic regions displaying conserved or newly formed interactions could identify new alterations involved in MCL pathogenesis.

### Translocations induce large-scale gene expression effects on derivative 14

**Figure 4.**
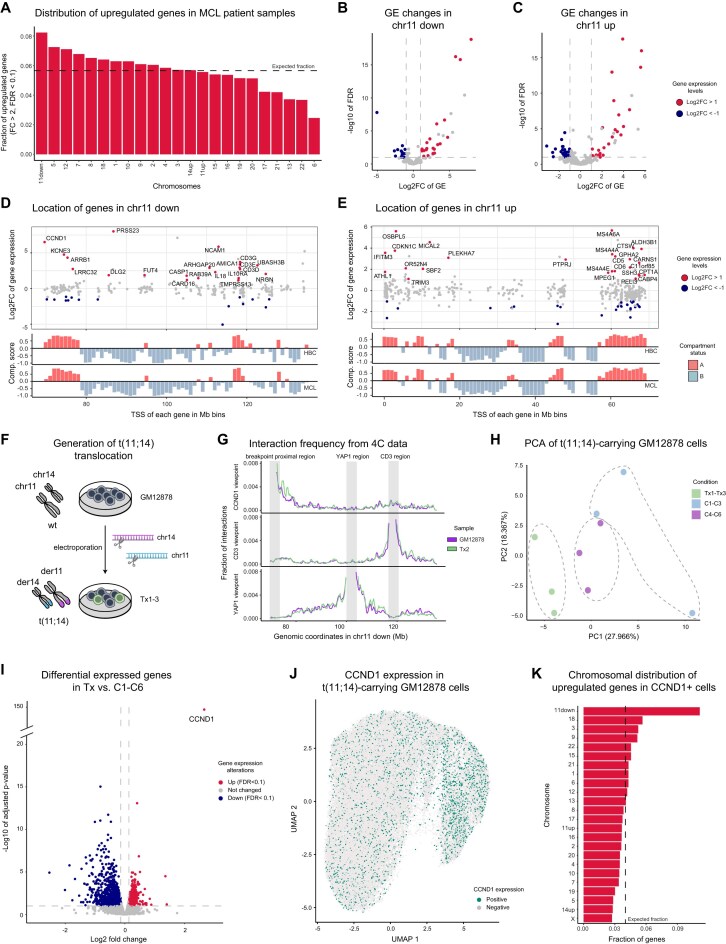
Gene expression analysis in cells carrying translocation t(11;14). (**A**) Fraction of upregulated protein-coding genes in MCL patients versus mature healthy B cells per chromosome. The dashed line represents the expected fraction. (**B**) Volcano plot of protein-coding genes in chr11 down. The dashed lines show the absolute log2 FC threshold of 1, and the –log10 value of FDR 0.1. (**C**) As in B, but for chr11 up. (**D**) Location of up- and downregulated genes in MCL patients on chr11 down. Names for upregulated genes are depicted. Below, compartment scores of each Mb bin in MCL and healthy B cells are shown. (**E**) As D, but for chr11 up. (**F**) Scheme of in vitro translocation generation. (**G**) Interaction frequencies from different viewpoints to chr11 down measured by 4C-seq. Vertical bars indicate the breakpoint proximal (chr11: 69 500 000 – 73 500 000), YAP1 (chr11: 100 000 000 – 104 000 000), and CD3 (chr11: 116 500 000 – 120 500 000) region. (**H**) PCA of samples harboring translocations generated in vitro, and (**I**) volcano plot of gene expression changes, both based on bulk RNA-seq. (**J**) UMAP of samples harboring translocations generated in vitro, based on scRNA-seq. (**K**) Fraction of upregulated protein-coding genes per chromosome in CCND1-positive versus -negative cells, after in vitro induction of translocations. The dashed line represents the expected fraction. wt = wild-type, down = downstream of the breakpoint, up = upstream of the breakpoint, MCL = mantle cell lymphoma, HBC = healthy B cells.

In order to assess the functional effects associated with the t(11;14) translocation, we next analyzed published RNA-seq data to evaluate gene expression changes in MCL patients compared to healthy B cells [24]. Surprisingly, we detected that chr11 down contained the highest enrichment of MCL-specific upregulated protein-coding genes (FC 2, FDR < 0.1, Fig. 4A–C, Supplementary Table S8). Of note, in this analysis we focused only on those genes expressed in more than half of the MCL patients—representing the vast majority of the upregulated genes—to elucidate global transcription alterations in the presence of the translocation, rather than potential patient-specific effects. Nevertheless, we recognize that the latter could also relate to MCL development. Next, we observed that the upregulated genes were not randomly distributed over chr11 down. As expected, a subset of these genes was located close to the breakpoint (Fig. 4d). Interestingly however, we also detected a group of upregulated genes located approximately 50 Mb away from the breakpoint—around Mb bins 118–120—that get in close proximity to the IGH enhancer in 3D space upon translocation formation (Fig. 3A and C). A permutation test confirmed that this distribution was indeed not random, showing an enrichment of MCL upregulated genes in Mb bin 118–120 (*P*-value 0.0402, Supplementary Table S9). Furthermore, other regions showing a significant enrichment of upregulated genes were located at Mb bin 106–108 on chr11 down (*P*-value 0.0214). Based on these results, we hypothesize that IGH enhancer hijacking by distant genes located on chr11 down may occur due to ultra-long-range interactions. On the other hand, we could not identify a global enrichment of upregulated genes in chr11 up and chr14 up (Fig. 4A, Supplementary Fig. S4A). However, upregulated genes on chr11 up were grouped close to the telomere and at the breakpoint region (Fig. 4E), with Mb bin 67–69 clearly enriched for upregulated genes (*P*-value 0.0278, Supplementary Table S9). Overall, we hypothesize based on this data that the shift of derivative 11 toward the nuclear center may favor the upregulation of these genes.

Gene ontology analyses suggested a significant enrichment of T-cell-related pathways, as well as apoptotic- and necroptotic-related gene networks (Supplementary Fig. S4B–E) in upregulated genes on chr11 down and chr14 up, respectively. Of note, Mb bin 118–120 on chromosome 11 harbors the *CD3* Epsilon, Delta and Gamma subunits, *AMICA1, IL10RA* and *TMPRSS13*. While the *CD3* genes encode for the different subunits of the T-cell receptor CD3 complex, IL10RA and TMPRSS13 are related to MAPK and TGF-beta signaling pathways, and AMICA1 controls the activation and migration of leukocytes. Gene ontology analyses of upregulated genes in chr11 up did not detect any enrichment of specific pathways. Nevertheless, we want to highlight the upregulation of CD5, MS4A paralogs and CTSW, among others, which are located at Mb bins 60–69, close to the breakpoint region (Fig. 4E). CD5 is a known surface marker present on MCL cells, and MS4A4 and MS4A6 are associated with macrophage-related immune responses, while CTSW regulates T-cell cytolytic activity.

The transcriptional changes described above are intriguing. However, whether they are a direct consequence of the translocation cannot be concluded from RNA-seq analysis in patient samples. In order to prove the relationship between the observed gene expression changes and the presence of the t(11;14) translocation, we generated the t(11;14) translocation *de novo* in the lymphoblastoid B cell line GM12878. To that end, we nucleofected GM12878 cells with ribonucleoprotein complexes (RNPs) targeting regions on chromosomes 11 and 14 close to the translocation breakpoints described in MCL patients (Fig. 4F). As a consequence of the induced double-strand breaks, the t(11;14) translocation was formed in a subset of cells as confirmed by karyotyping, and estimated to represent 10–12% of the entire population by digital PCR (Supplementary Fig. S4F). As before, 4C-seq-based interaction frequencies indicated the presence of the ultra-long-range interaction between the CD3 locus and the breakpoint region in the engineered cell lines carrying the t(11;14) translocation (Fig. 4G). We furthermore detected an approximately 9-fold increase in CCND1 levels by qPCR in these heterogeneous populations compared to the controls (nucleofected with only one gRNA on chromosome 11 or 14), showing that the presence of 10–12% of cells with translocations is enough to identify strongly upregulated transcripts (Supplementary Fig. S4G). Next, we performed bulk RNA-seq of the populations harboring cells with the translocation, as well as the control populations. We could clearly distinguish them by PCA (Fig. 4H) and identified 1086 upregulated and 1206 downregulated genes (FC 1.1, FDR < 0.1, Fig. 4I, Supplementary Fig. S4H, Supplementary Table S10). Although we did not see a clear enrichment of upregulated genes on chr11 down, it still harbored more upregulated genes than expected by a random distribution (Supplementary Fig. S4I). Moreover, the permutation test pointed again toward a non-random distribution of bins with an enrichment of upregulated genes, located at Mb bin 118–120 (*P*-value 0.0554), as well as to an enrichment of upregulated genes on chr11 up, bin 10–12 and bin 61–63 (*P*-values 0.0022 and 0.0013, respectively) (Supplementary Table S9).

To further investigate translocation-specific effects, we performed single-cell RNA-seq analyses. As expected, we detected approximately 11% of CCND1-positive cells in our samples, which we considered a proxy for the presence of the induced translocation. Interestingly, they do not cluster separately from CCND1-negative cells, suggesting that there is a high level of similarity between the CCND1-positive and -negative populations, possibly combined with substantial heterogeneity within the CCND1-positive population (Fig. 4J). Nevertheless, the upregulated genes in CCND1-positive cells showed a strong overrepresentation of genes on chr11 down (Fig. 4K, Supplementary Table S11), which was not observed for any other chromosome. These results highly suggest that the upregulation of these genes is linked to the presence of the translocation. More detailed analysis showed an increased number of upregulated genes in Mb bins 82–84 and bins 118–120 on chr11 down (respective p-values 0.0335 and 0.0594), and in Mb bins 64–66 (*P*-value 0.0287) on chr11 up (Supplementary Table S9). The latter two regions are in line with our previous data in MCL patients. Of note, the actual upregulated genes on chromosome 11 do not overlap between MCL patients and our *in vitro* translocation model (Supplementary Fig. S4J). Thus, while the affected genomic regions are the same at the Mb scale, the genes with altered expression levels are different. To better understand the role of the IGH enhancer on chr11 down, we calculated the correlation between the gene expression fold change and the 3D distance to the translocation breakpoint region, which contains the IGH enhancer. Interestingly, we observed a significant negative correlation, showing that genomic regions closer to the IGH enhancer undergo stronger gene expression effects. Importantly, this correlation was not observed when using the linear distance to the breakpoint (Supplementary Fig. S4K). Finally, we generated high resolution single-cell SMART-seq2 data to study allele-specific effects and show that, when the translocation involves the maternal allele of chr11 down (*n* = 50 cells versus 701 cells without translocations), a clear increase in maternal transcripts is observed. Surprisingly, this allele-specific pattern was not observed for the paternal allele (*n* = 27 cells versus 701 cells without translocations), pointing toward possible allele-specific effects that warrant further investigation in follow-up studies (Supplementary Fig. S4L and Supplementary Table S12).

In conclusion, both in MCL patients and in *in vitro* translocation models we detect major gene expression alterations in the entire chromosomal segment of chr11 down, with prominent effects in regions that get closer to the IGH enhancer in 3D space. Based on these results, we consider that this entire chromosome arm, covering over 50 Mb, harbors a more permissive gene regulatory environment induced by the IGH enhancer. Interestingly, the large majority of upregulated genes on this chromosomal segment in our single-cell assay already contained an active promoter in wild-type GM12878 cells (90.2%, 46/51 genes, Supplementary Table S11), which is strikingly more than expected by chance (46.8%, 214/457 genes), suggesting that the IGH enhancer has limited capacity to change promoter state—except within its neo-TAD leading to CCND1 overexpression—but rather enhances gene expression of active genes. Interestingly, seven out of eight (87.5%) translocation-induced upregulated genes in Mb bin 64–66 on chr11 up, which is subject to a large change in radial positioning, also already displayed an active promoter state prior to translocation formation (Supplementary Table S11). Overall, these observations could explain why in MCL patients and in our *in vitro* translocation model different genes become upregulated upon translocation formation. More specifically, the active genes in precursor B cells, which acquire the translocation in MCL patients, are different from those that are active in the mature lymphoblastoid GM12878 cell line used in this study. Based on this, the translocation will induce different gene expression changes. Altogether our results stress the importance of the epigenetic state of the cell in which translocations occur, both at the 3D genome and genome activation level, as it will determine their downstream gene expression effects and thus their oncogenic transformation potential.

## Discussion

**Figure 5.**
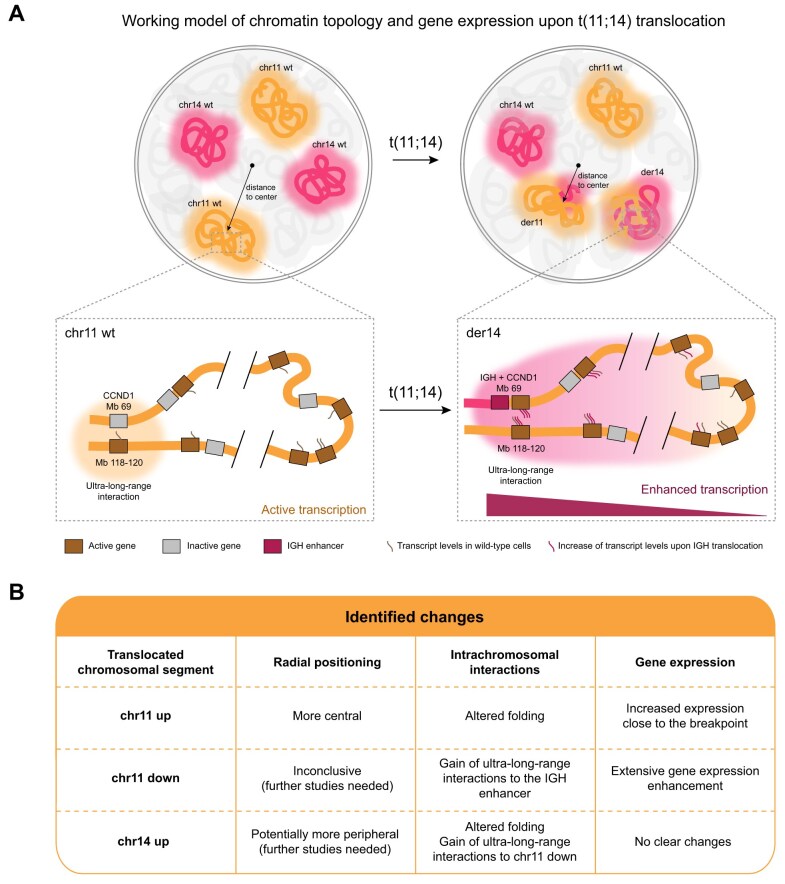
Working model. (**A**) Both translocated chromosomes, derivative 11 (der11) and derivative 14 (der14), formed upon the t(11;14) in MCL undergo genome organization changes compared to wild-type, with der11 moving toward the nuclear center and der14 showing *de novo* intrachromosomal interaction formation involving the IGH enhancer close and distant from the breakpoint. We furthermore show that the latter causes a pattern of enhanced transcription (indicated by additional mRNA transcripts) affecting the entire segment of chromosome 11 downstream of the breakpoint, with the bins around Mb 120 being most susceptible to upregulate gene expression. (**B**) Summary table of all identified genome organization and gene expression changes in the translocated segments of chr11 up (located on der11), chr11 down and chr14 up (both located on der14) compared to their wild-type counterparts.

It is commonly known that SVs, such as translocations, can play a key role in disease development, particularly in malignancies [10, 12, 55]. However, the complex genome-wide relationship between SVs, genome reorganization, and gene expression alterations in tumors remains largely unknown. In this work, we provide a detailed picture of the link between these layers in the context of MCL-associated translocations (Fig. 5). While 3C techniques, especially in combination with long-read sequencing, are utilized more and more to resolve complex karyotypes in cancer [56], our results demonstrate that *in vitro* SV generation and downstream analyses are key to provide a holistic view of the effects of SVs on genome regulation. This approach enabled us to identify consequences of IGH translocations beyond the limited effects reported so far, which only focus on single genes close to the breakpoint, such as *CCND1* in MCL, and *BCL6, BCL2* and *MYC* in other NHLs [18, 57–59]. Our single-cell analysis furthermore allowed us to distinguish cell intrinsic translocation-based effects from paracrine effects affecting the entire cellular pool. The former were largely masked in our bulk RNA-seq data, except for the non-random distribution of upregulated genes on chr11 up and down 11, highlighting the essence of mixed population-based single-cell analysis to identify them. Overall, empowering us to show that MCL-associated IGH translocations upregulate a large set of genes distributed over entire chromosome arms through different molecular mechanisms.

First of all, we unraveled that MCL cells present an altered CT positioning pattern compared to healthy B cells. In line with previous studies widely reporting that short and gene-rich chromosomes occupy more central nuclear positions [8, 60–62], we detected that derivative 11, which drastically decreases size and increases gene density, moves toward the nuclear center. Simultaneously, we show that its regions close to the breakpoint display an enriched number of upregulated genes in MCL patients (Mb bin 67–69). Our results are in agreement with Bintou *et al.* (BioRxiv, 2019) who reported that der11 moves toward the nuclear center, and detected an upregulation of more than 80 genes in chromosome 11, in four MCL patient samples compared to a healthy LCL cell line (LCL RPMI8866). Importantly, the findings of our *in vitro* translocation models go even beyond, showing that the translocation results in upregulation of genes on chr11 up close to the breakpoint (Mb bin 64–66). Notably, almost all translocation-induced upregulated genes in these bins harbored an active promoter prior to translocation formation.

Our genetically engineered cells furthermore unveiled that *de novo* translocations induce major gene expression upregulation in derivative 14 downstream of the translocation breakpoint. However, as for chr11 up, not all genes on this chromosomal segment become upregulated. Genes most susceptible to change expression are those located at Mb 118–120 on chr11 down, which are part of a conserved, ultra-long-range interaction spanning 50 Mb linking these genes to the *CCND1* locus in 3D space. Upon translocation formation, this conserved 3D structure leads to the establishment of ultra-long-range interactions between the strong IGH enhancer and the cluster of genes mentioned above. In line with this, we show that the proximity to the IGH enhancer correlates with the level of gene expression enhancement in chr11 down. Importantly though, the induced gene expression effect is largely limited to genes with active promoters prior to translocation induction, highlighting that close proximity to the strong IGH enhancer alone is not sufficient to alter gene expression. *CCND1*, located next to the IGH enhancer within the neo-TAD, is an exception to this rule, changing its promoter activity and expression upon translocation formation. Interestingly, we show that the translocation-induced expression effects are not limited to the highly susceptible region on chr11 down, but can extend to genes on this entire chromosome arm. Further research is necessary to explore whether all genes in this segment are simultaneously upregulated in individual cells or whether heterogeneity exists. Our single-cell analysis points to the latter, although the intrinsic sparsity of this data does not allow us to draw definitive conclusions. To the best of our knowledge, this represents a so far unreported phenomenon in which cancer cells utilize translocations to hijack existing ultra-long-range interactions over tens of megabases to alter gene expression.

Our findings have major consequences for understanding tumor formation, since they show that functional effects of translocations depend on pre-existing epigenetic characteristics such as genome organization and chromatin states, to induce oncogenic effects. This finding is particularly important in the current era in which single-cell approaches enable the identification of more and more cell subtypes. Our results will thus facilitate the prediction of translocation-induced effects in known and new cell subtypes, allowing us to better pinpoint tumoral cells of origin. In MCL patients in particular, translocations occur in early B cells, but they do not fully transform into MCL cells until they reach the naive B cell stage. Our observations open doors to hypothesize that the genome activity state of naive B cells may provide key triggers to upregulate specific genes on chromosome 11 that drive tumor development. Another important observation with relevance for tumor formation is the allele-specific effect of translocations. Providing further understanding of how translocations can establish differential effects on maternal and paternal translocation partners, potentially related to allele-specific 3D folding, will be key to gain deeper insights into tumorigenesis.

Finally, our study provides a clear set of genes to be explored for their oncogenic effects in MCL, as a solid basis to design better murine tumor models and therapeutic strategies in the context of MCL that is so far considered incurable. Upregulated genes in Mb bin 118–120 on chromosome 11, for example, emphasize the activation of T-cell pathways in MCL cells, including upregulation of CD3. While the expression of the T-cell marker CD5 is a common feature in MCL, cases with cytoplasmic expression of CD3 have been only sporadically reported [63, 64]. Interestingly, T/B biphenotypic lymphocytes, expressing both CD5 and CD3 and originating from a subset of mature B cells, were recently found by Zhang *et al.* [65] in healthy murine and human blood. CD5+/CD3 + cells have furthermore been reported to represent B-1a cells related to autoimmune disease [66]. Altogether, these results might indicate a link between T/B biphenotypic lymphocytes, CD5+/CD3 + B-1a cells and CD5+/CD3 + MCL cells. However, follow-up studies are needed to investigate whether these cells could be related to the cell of origin of MCL or how T-cell activation by other means could contribute to MCL pathogenesis.

In conclusion, the novel, broad-reaching changes of IGH translocations in genome organization, together with the associated transcriptional alterations, emphasize the importance of genomic integrity to prevent disease development. Future research will reveal similar mechanisms by which ultra-long-range interactions shape the gene expression landscape in healthy tissues, and show how a broad range of SVs can influence this phenomenon to drive tumor development. These findings will be crucial for a better understanding of gene regulation as a whole in the context of both health and disease. In the specific context of cancer, they will unveil new mechanisms of early carcinogenesis, paving the way for developing more accurate pre-clinical tumor models, more precise diagnostic tools, and new therapeutic strategies, due to a better global understanding of the early onset of malignant transformation.

## Supplementary Material

gkaf677_Supplemental_Files

## Data Availability

Hi-C and RNA-seq data of patient samples and healthy donors were mined from a previous study [24] and can be found at the European Genome-Phenome Archive (EGA, http://www.ebi.ac.uk/ega/), which is hosted at the European Bioinformatics Institute (EBI), accession number EGAS00001004763 and EGAS00001000327. SNP6.0 array data of the MCL cell line Z-138, generated in a previous study [28], was submitted to GEO (record GSE278105). Hi-C, Tiled-C, 4C-seq, and chromosome sorting data of cell lines, as well as bulk and single-cell (Singleron and SMART-seq2) gene expression data upon translocation formation in GM12878, all generated in this study, have been deposited in SRA (BioProject number PRJNA853734). Imaging data using break break-apart probes in different cell lines, generated in this study, have been deposited to the BioImage Archive (Accession number S-BIAD1401).

## References

[1] Cremer T, Cremer C Chromosome territories, nuclear architecture and gene regulation in mammalian cells. Nat Rev Genet. 2001; 2:292–301. 10.1038/35066075. 11283701

[2] Boyle S, Gilchrist S, Bridger JM et al. The spatial organization of human chromosomes within the nuclei of normal and emerin-mutant cells. Hum Mol Genet. 2001; 10:211–9. 10.1093/hmg/10.3.211. 11159939

[3] Fudenberg G, Imakaev M, Lu C et al. Formation of chromosomal domains by loop extrusion. Cell Rep. 2016; 15:2038–49. 27210764 10.1016/j.celrep.2016.04.085 PMC4889513

[4] Rao SSP, Huntley MH, Durand NC et al. A three-dimensional map of the human genome at kilobase resolution reveals principles of chromatin looping. Cell. 2014; 159:1665–80. 10.1016/j.cell.2014.11.021. 25497547 PMC5635824

[5] Dixon JR, Selvaraj S, Yue F et al. Topological domains in mammalian genomes identified by analysis of chromatin interactions. Nature. 2012; 485:376–80. 10.1038/nature11082. 22495300 PMC3356448

[6] Aiden EL, Van Berkum NL, Williams L et al. Comprehensive mapping of long range interactions reveals folding principles of the human genome. Science. 2009; 326:289–93. 10.1126/science.1181369. 19815776 PMC2858594

[7] Tavares-Cadete F, Norouzi D, Dekker B et al. Multi-contact 3C reveals that the human genome during interphase is largely not entangled. Nat Struct Mol Biol. 2020; 27:1105–14. 10.1038/s41594-020-0506-5. 32929283 PMC7718335

[8] Girelli G, Custodio J, Kallas T et al. GPSeq reveals the radial organization of chromatin in the cell nucleus. Nat Biotechnol. 2020; 38:1184–93. 10.1038/s41587-020-0519-y. 32451505 PMC7610410

[9] Guelen L, Pagie L, Brasset E et al. Domain organization of human chromosomes revealed by mapping of nuclear lamina interactions. Nature. 2008; 453:948–51. 10.1038/nature06947. 18463634

[10] Nowell PC, Hungerford DA Chromosome studies in normal and leukemic Human leukocytes. J Natl Cancer Inst. 1960; 25:85–109. 14427847

[11] Groffen J, Stephenson JR, Heisterkamp N et al. Philadelphia chromosomal breakpoints are clustered within a limited region, bcr, on chromosome 22. Cell. 1984; 36:93–9. 10.1016/0092-8674(84)90077-1. 6319012

[12] de Thé H, Lavau C, Marchio A et al. The PML-RARα fusion mRNA generated by the t(15;17) translocation in acute promyelocytic leukemia encodes a functionally altered RAR. Cell. 1991; 66:675–84. 10.1016/0092-8674(91)90113-D. 1652369

[13] Grosveld G, Verwoerd T, van Agthoven T et al. The chronic myelocytic cell line K562 contains a breakpoint in bcr and produces a chimeric bcr /c - abl transcript. Mol Cell Biol. 1986; 6:607–16. 3023859 10.1128/mcb.6.2.607-616.1986 PMC367552

[14] Gröschel S, Sanders MA, Hoogenboezem R et al. A single oncogenic enhancer rearrangement causes concomitant EVI1 and GATA2 deregulation in Leukemia. Cell. 2014; 157:369–81. 10.1016/j.cell.2014.02.019. 24703711

[15] Taslerová R, Kozubek S, Bártová E et al. Localization of genetic elements of intact and derivative chromosome 11 and 22 territories in nuclei of Ewing sarcoma cells. J Struct Biol. 2006; 155:493–504. 10.1016/j.jsb.2006.05.005. 16837212

[16] Taslerová R, Kozubek S, Lukášová E et al. Arrangement of chromosome 11 and 22 territories, EWSR1 and FLI1 genes, and other genetic elements of these chromosomes in human lymphocytes and Ewing sarcoma cells. Hum Genet. 2003; 112:143–55. 10.1007/s00439-002-0847-7. 12522555

[17] Zhegalova IV, Vasiluev PA, Flyamer IM et al. Trisomies reorganize Human 3D genome. Int J Mol Sci. 2023; 24:16044 10.3390/ijms242216044. 38003233 PMC10671006

[18] Küppers R Mechanisms of B-cell lymphoma pathogenesis. Nat Rev Cancer. 2005; 5:251–62. 10.1038/nrc1589. 15803153

[19] Jares P, Colomer D, Campo E Genetic and molecular pathogenesis of mantle cell lymphoma: perspectives for new targeted therapeutics. Nat Rev Cancer. 2007; 7:750–62. 10.1038/nrc2230. 17891190

[20] Silkenstedt E, Dreyling M Mantle cell lymphoma—Update on molecular biology, prognostication and treatment approaches. Hematol Oncol. 2023; 41:36–42. 10.1002/hon.3149. 37294961

[21] Navarro A, Beà S, Jares P et al. Molecular pathogenesis of mantle cell lymphoma. Hematol Oncol Clin North Am. 2020; 34:795–807. 10.1016/j.hoc.2020.05.002. 32861278 PMC7473344

[22] Bodrug SE, Warner BJ, Bath ML et al. Cyclin D1 transgene impedes lymphocyte maturation and collaborates in lymphomagenesis with the myc gene. EMBO J. 1994; 13:2124–30. 10.1002/j.1460-2075.1994.tb06488.x. 8187765 PMC395065

[23] Lovec H, Grzeschiczek A, Kowalski MB et al. Cyclin D1/bcl-1 cooperates with myc genes in the generation of B-cell lymphoma in transgenic mice. EMBO J. 1994; 13:3487–95. 10.1002/j.1460-2075.1994.tb06655.x. 8062825 PMC395252

[24] Vilarrasa-Blasi R, Soler-Vila P, Verdaguer-Dot N et al. Dynamics of genome architecture and chromatin function during human B cell differentiation and neoplastic transformation. Nat Commun. 2021; 12:1–18. 10.1038/s41467-020-20849-y. 33510161 PMC7844026

[25] Kuderna LFK, Lizano E, Julià E et al. Selective single molecule sequencing and assembly of a human Y chromosome of African origin. Nat Commun. 2019; 10:4 10.1038/s41467-018-07885-5. 30602775 PMC6315018

[26] Orlando G, Kinnersley B, Houlston RS Capture hi-C library generation and analysis to detect chromatin interactions. CP Human Genetics. 2018; 98:e63 10.1002/cphg.63. 29979818

[27] Serra F, Baù D, Goodstadt M et al. Automatic analysis and 3D-modelling of Hi-C data using TADbit reveals structural features of the fly chromatin colors. PLoS Comput Biol. 2017; 13:1–17. 10.1371/journal.pcbi.1005665. PMC5540598 28723903

[28] Beà S, Valdés-Mas R, Navarro A et al. Landscape of somatic mutations and clonal evolution in mantle cell lymphoma. Proc Natl Acad Sci USA. 2013; 110:18250–5. 10.1073/pnas.1314608110. 24145436 PMC3831489

[29] Splinter E, de Wit E, van de Werken HJG et al. Determining long-range chromatin interactions for selected genomic sites using 4C-seq technology: from fixation to computation. Methods. 2012; 58:221–30. 10.1016/j.ymeth.2012.04.009. 22609568

[30] Danecek P, Bonfield JK, Liddle J et al. Twelve years of SAMtools and BCFtools. Gigascience. 2021; 10:giab008 10.1093/gigascience/giab008. 33590861 PMC7931819

[31] Quinlan AR, Hall IM BEDTools: a flexible suite of utilities for comparing genomic features. Bioinformatics. 2010; 26:841–2. 10.1093/bioinformatics/btq033. 20110278 PMC2832824

[32] Beekman R, Chapaprieta V, Russiñol N et al. The reference epigenome and regulatory chromatin landscape of chronic lymphocytic leukemia. Nat Med. 2018; 24:868–80. 10.1038/s41591-018-0028-4. 29785028 PMC6363101

[33] Oudelaar AM, Beagrie RA, Gosden M et al. Dynamics of the 4D genome during in vivo lineage specification and differentiation. Nat Commun. 2020; 11:2722 10.1038/s41467-020-16598-7. 32483172 PMC7264236

[34] Downes DJ, Smith AL, Karpinska MA et al. Capture-C: a modular and flexible approach for high-resolution chromosome conformation capture. Nat Protoc. 2022; 17:445–75. 10.1038/s41596-021-00651-w. 35121852 PMC7613269

[35] Imakaev M, Fundenberg G, McCord RP et al. Iterative correction of hi-C data reveals hallmarks of. Nature. 2012; 9:999–1003. 10.1038/nmeth.2148 PMC3816492 22941365

[36] Kozubek M, Matula P An efficient algorithm for measurement and correction of chromatic aberrations in fluorescence microscopy. J Microsc. 2000; 200:206–17. 10.1046/j.1365-2818.2000.00754.x. 11106961

[37] Besag J On the statistical analysis of dirty pictures. J R Stat Soc Ser B Stat Methodol. 1986; 48:259–79. 10.1111/j.2517-6161.1986.tb01412.x.

[38] Cousty J, Bertrand G, Najman L et al. Watershed cuts: minimum spanning forests and the drop of water principle. IEEE Trans Pattern Anal Mach Intell. 2009; 31:1362–74. 10.1109/TPAMI.2008.173. 19542572

[39] Meijster A, Roerdink J, Hesselink W A general algorithm for computing distance transforms in linear time. Math Morphol Its Appl to Image Signal Procesing. 2000; Boston Kluwer Acad Publ 331–40.

[40] Paulsen J, Sekelja M, Oldenburg AR et al. Chrom3D: three-dimensional genome modeling from hi-C and nuclear lamin-genome contacts. Genome Biol. 2017; 18:21 10.1186/s13059-016-1146-2. 28137286 PMC5278575

[41] Ecker S, Chen L, Pancaldi V et al. Genome-wide analysis of differential transcriptional and epigenetic variability across human immune cell types. Genome Biol. 2017; 18:18 10.1186/s13059-017-1156-8. 28126036 PMC5270224

[42] Love MI, Huber W, Anders S Moderated estimation of fold change and dispersion for RNA-seq data with DESeq2. Genome Biol. 2014; 15:550 10.1186/s13059-014-0550-8. 25516281 PMC4302049

[43] Yu G, Wang LG, Han Y et al. ClusterProfiler: an R package for comparing biological themes among gene clusters. OMICS. 2012; 16:284–7. 10.1089/omi.2011.0118. 22455463 PMC3339379

[44] Chen EY, Tan CM, Kou Y et al. Enrichr: interactive and collaborative HTML5 gene list enrichment analysis tool. BMC Bioinf. 2013; 14:128 10.1186/1471-2105-14-128. PMC3637064 23586463

[45] Kuleshov MV, Jones MR, Rouillard AD et al. Enrichr: a comprehensive gene set enrichment analysis web server 2016 update. Nucleic Acids Res. 2016; 44:W90–7. 10.1093/nar/gkw377. 27141961 PMC4987924

[46] Picelli S, Björklund ÅK, Faridani OR et al. Smart-seq2 for sensitive full-length transcriptome profiling in single cells. Nat Methods. 2013; 10:1096–8. 10.1038/nmeth.2639. 24056875

[47] Severino J, Bauer M, Mattimoe T et al. Controlled X-chromosome dynamics defines meiotic potential of female mouse in vitro germ cells. EMBO J. 2022; 41:e109457 10.15252/embj.2021109457. 35603814 PMC9194795

[48] Rozowsky J, Abyzov A, Wang J et al. AlleleSeq: analysis of allele-specific expression and binding in a network framework. Mol Syst Biol. 2011; 7:522. 21811232 10.1038/msb.2011.54 PMC3208341

[49] Dobin A, Davis CA, Schlesinger F et al. STAR: ultrafast universal RNA-seq aligner. Bioinformatics. 2013; 29:15–21. 10.1093/bioinformatics/bts635. 23104886 PMC3530905

[50] van de Geijn B, McVicker G, Gilad Y et al. WASP: allele-specific software for robust molecular quantitative trait locus discovery. Nat Methods. 2015; 12:1061–3. 10.1038/nmeth.3582. 26366987 PMC4626402

[51] Tanabe H, Habermann FA, Solovei I et al. Non-random radial arrangements of interphase chromosome territories: evolutionary considerations and functional implications. Mutat Res. 2002; 504:37–45. 10.1016/S0027-5107(02)00077-5. 12106644

[52] Bolzer A, Kreth G, Solovei I et al. Three-dimensional maps of all chromosomes in human male fibroblast nuclei and prometaphase rosettes. PLoS Biol. 2005; 3:0826–42. 10.1371/journal.pbio.0030157. PMC1084335 15839726

[53] Tanabe H, Müller S, Neusser M et al. Evolutionary conservation of chromosome territory arrangements in cell nuclei from higher primates. Proc Natl Acad Sci USA. 2002; 99:4424–9. 10.1073/pnas.072618599. 11930003 PMC123664

[54] Estrov Z, Talpaz M, Ku S et al. Z-138: a new mature B-cell acute lymphoblastic leukemia cell line from a patient with transformed chronic lymphocytic leukemia. Leuk Res. 1998; 22:341–53. 10.1016/S0145-2126(97)00191-4. 9669839

[55] Nambiar M, Kari V, Raghavan SC Chromosomal translocations in cancer. Biochim Biophys Acta (BBA). 2008; 1786:139–52. 10.1016/j.bbcan.2008.07.005. 18718509

[56] Mallard C, Johnston MJ, Bobyn A et al. Hi-C detects genomic structural variants in peripheral blood of pediatric leukemia patients. Cold Spring Harb Mol Case Stud. 2022; 8:a006157 10.1101/mcs.a006157. 34819303 PMC8744495

[57] Galteland E, Sivertsen EA, Svendsrud DH et al. Translocation t(14;18) and gain of chromosome 18/BCL2: effects on BCL2 expression and apoptosis in B-cell non-hodgkin's lymphomas. Leukemia. 2005; 19:2313–23. 10.1038/sj.leu.2403954. 16193090

[58] Ye BH, Chaganti S, Chang CC et al. Chromosomal translocations cause deregulated BCL6 expression by promoter substitution in B cell lymphoma. EMBO J. 1995; 14:6209–17. 10.1002/j.1460-2075.1995.tb00311.x. 8557040 PMC394745

[59] Ott G, Rosenwald A, Campo E Understanding MYC-driven aggressive B-cell lymphomas: pathogenesis and classification. 2013; 122:575–83. 10.1182/asheducation-2013.1.575 24319234

[60] Cremer M, Hase JV, Volm T et al. Non-random radial higher-order chromatin arrangements in nuclei of diploid human cells. Chromosom Res. 2001; 9:541–67. 10.1023/A:1012495201697. 11721953

[61] Croft JA, Bridger JM, Boyle S et al. Differences in the localization and morphology of chromosomes in the human nucleus. J Cell Biol. 1999; 145:1119–31. 10.1083/jcb.145.6.1119. 10366586 PMC2133153

[62] Sun HB, Shen J, Yokota H Size-dependent positioning of human chromosomes in interphase nuclei. Biophys J. 2000; 79:184–90. 10.1016/S0006-3495(00)76282-5. 10866946 PMC1300924

[63] Felten CL, Chan JA, DeCastro DR et al. Blastoid variant mantle cell lymphoma expressing aberrant CD3 and CD10 with concurrent small lymphocytic lymphoma: establishment of a clonal relationship by B- and T-cell receptor gene rearrangements. Case Rep Hematol. 2018; 2018:8303571 10.1155/2018/8303571. 30627460 PMC6305046

[64] Malek A, Bhagat G Cyclin D1−negative mantle cell lymphoma with aberrant CD3 expression. Blood. 2017; 130:1388 10.1182/blood-2017-05-783274. 28912297

[65] zhang Y, Guo C, Zhou Y et al. A biphenotypic lymphocyte subset displays both T- and B-cell functionalities. Commun Biol. 2024; 7:28. 38182721 10.1038/s42003-023-05719-9 PMC10770049

[66] Yamamoto W, Toyoda H, Xu DQ et al. CD3+ B-1a cells as a mediator of disease progression in autoimmune-prone mice. Mediat Inflamm. 2018; 2018:9289417 10.1155/2018/9289417. PMC6323491 30670930

