# Supplementary material for: Translocations can drive expression changes of multiple genes in regulons covering entire chromosome arms
Source: Nucleic Acids Res. 2025 Aug 22;53(15):gkaf677. doi: 10.1093/nar/gkaf677 (PMC12370299; doi:10.1093/nar/gkaf677)
Supplement: gkaf677_Supplemental_Files [file gkaf677_supplemental_files.zip › Supplementary_data_for_resubmission_final.pdf]

Supplementary data belonging to manuscript by Oncins *et al*, entitled:

Translocations can drive expression changes of multiple genes in regulons covering entire chromosome arms.

Contents

Supplementary Table Legends..Page 2

Supplementary Figure 1..Page 3

Supplementary Figure 2..Page 4

Supplementary Figure 3..Page 6

Supplementary Figure 4..Page 8

Supplementary Table Legends

Supplementary Table 1. Sample characteristics. Characteristics of the primary samples used in the study as well as the reference to previous studies in which the samples were used. MCL = mantle cell lymphoma, CLL = chronic lymphocytic leukemia, NA = non-assigned.

Supplementary Table 2. Heterozygous SNPs in MCL cell line Z-138. Full list of heterozygous SNPs on chromosomal segments chr11 up, chr11 down and chr14 up, including genotype information on the non-translocated - not involving the t(11;14) - and translocated - involving the t(11;14) – alleles.

Supplementary Table 3. IGH translocation breakpoints in cell lines. IGH translocation breakpoints or IGH insertion locations in Z-138, JVM-2, U-266, KARPAS422 and Pfeiffer cell lines, defined using the Tiled-C data at 5 kb resolution.

Supplementary Table 4. Break-apart probes. Characteristics of break-apart probes targeting the *CCND1* and *IGH* loci for FISH experiments.

Supplementary Table 5. Structural variants in MCL samples. List of patient-specific structural variants found in the MCL patients included in this study, as defined by Nadeu *et al*, Blood 2020 (WGS) and Beà *et al*, PNAS 2013 (WGS, WES, SNP arrays).

Supplementary Table 6. Common altered interchromosomal interactions in MCL. List of common top 10% of interchromosomal interactions altered in MCL versus healthy B cells and CLL.

Supplementary Table 7. Number of allele-specific reads on chromosomes 11 and 14 in Z-138. Number of Hi-C reads in Z-138 that map to either the non-translocated - not involving the t(11;14) - and translocated - involving the t(11;14) – chromosome 11 or 14, representing an interchromosomal ligation product.

Supplementary Table 8. Differentially expressed genes in MCL samples compared to healthy B cells. Differentially expressed protein-coding genes in MCL compared to mature healthy B cells, absolute log2FC > 1, FDR < 0.1. Upregulated genes are expressed in at least 3 MCL patients.

Supplementary Table 9. Permutation test results for upregulated gene enrichment over 3MB bins. P-values from the permutation test regarding the number of upregulated genes per 3MB bins in our different models (MCL patients data, and bulk and scRNA-seq of the in vitro translocation generation models) versus the global expected background.

Supplementary Table 10. Differentially expressed genes upon *in vitro* translocation generation bulk. Differentially expressed protein-coding genes in GM12878 populations carrying ~10% translocated cells versus controls, absolute log2FC > log2(1.1), FDR < 0.1.

Supplementary Table 11. Differentially expressed genes upon *in vitro* translocation generation single-cell. Differentially expressed protein-coding genes in CCND1-positive GM12878 cells upon *in vitro* translocation generation, compared to CCND1-negative cells using scRNA-seq, FDR < 0.1.

Supplementary Table 12. Allele-specific read counts SMART-seq2 data. Number of allele-specific reads of genes on chr11 down that are upregulated upon *in vitro* translocation generation, comparing cells without translocations with those showing the IGH translocation to the maternal or paternal alelle of chromosome 11.

250307_chr11.alleseq.GIAB.overlap.GM12878.vcf. Haplotypes of chr11 in GM12878 using phased heterozygous SNP data (hg38), based on the overlap between the Genome in a Bottle Consortium (GIAB) and Rozowsky et al, J. Molecular systems biology. 2011;7:522.

Supplementary Figures

Supplementary figure 1

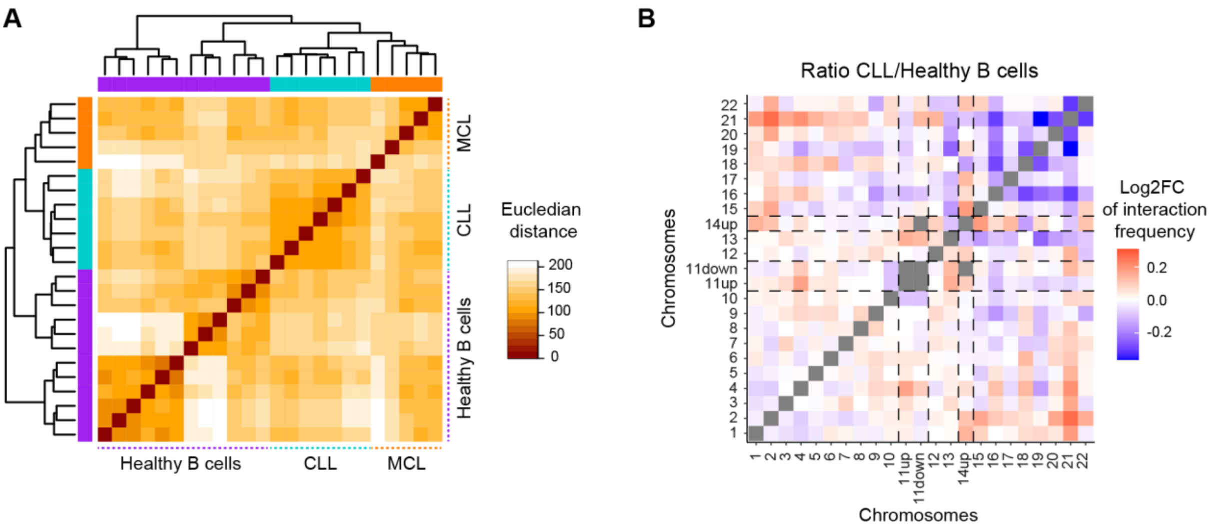

Supplementary Figure 1. The interchromosomal 3D landscape in MCL. A) Dissimilarity matrix comparing the Hi-C matrices of interchromosomal reads among samples, excluding reads between translocated segments (chr11 down with chr14 up; interactions between chr11up with chr14 down were neither included because chr14 down was removed from all analyses due to low Hi-C read numbers – see also main text). **B)** Log2 fold-change of the interaction frequency ratio between CLL and healthy B cells. Dashed lines highlight translocated chromosomal segments, divided into “up” and “down” as outlined in figure 1A. down = downstream of the breakpoint position, up = upstream of the breakpoint position.

Supplementary figure 2

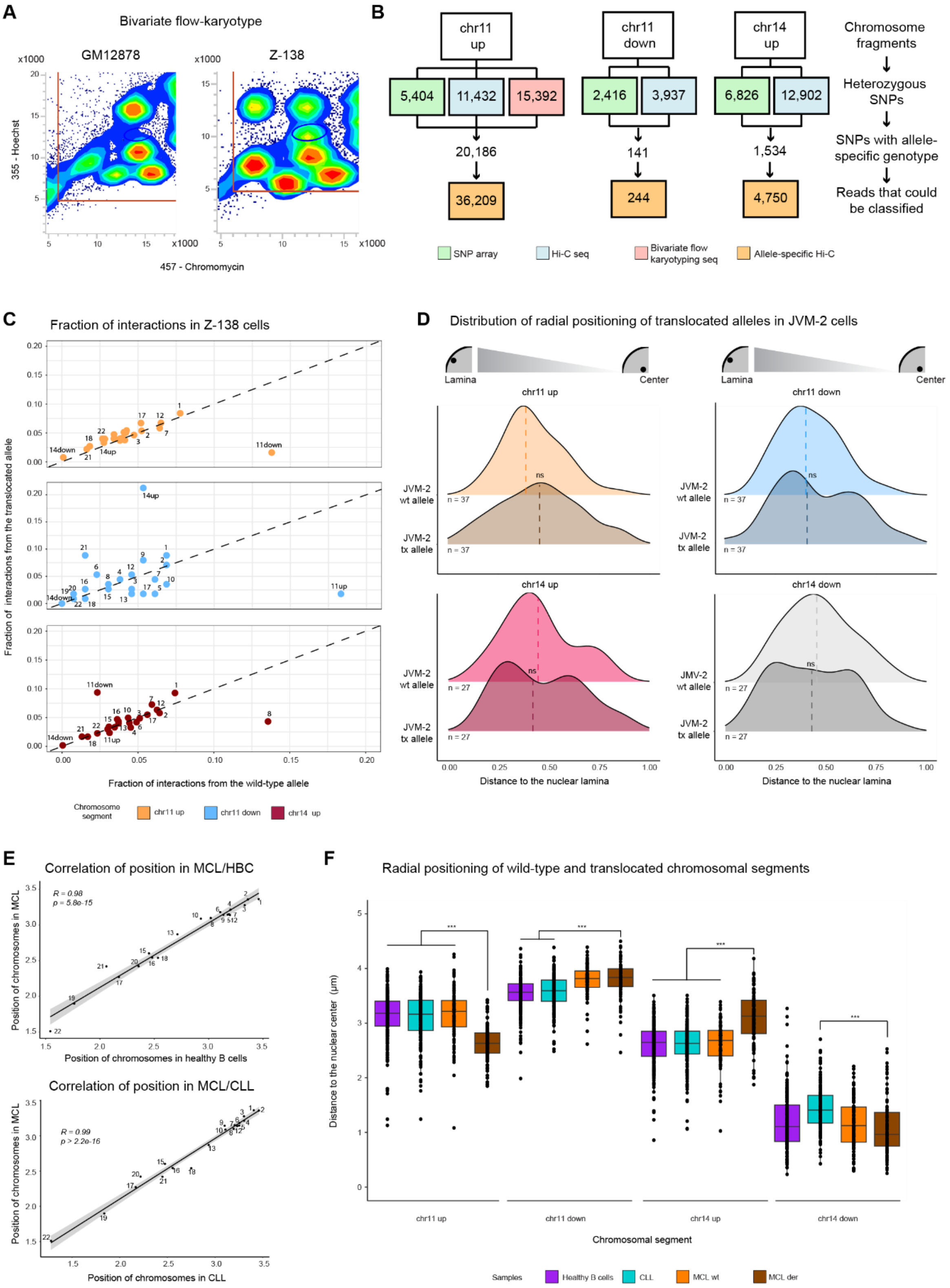

Supplementary Figure 2. Allele-specific analysis of translocated chromosomes in MCL. A) Bivariate flow karyotype of GM12878 and Z-138 cells showing different chromosome clouds, including the cloud representing der11 in Z-138, indicated by the blue oval, which is not present in GM12878 cells. The other differential cloud, present in the upper left quadrant in the male-derived Z-138 cell line represents chromosome Y, which is not present in the female-derived cell line GM12878. **B)** Flowchart of SNP classification per chromosomal segment. SNP array, Hi-C and bivariate flow karyotyping

sequencing data were used to identify heterozygous SNPs that were further used to genotype wild-type and translocated alleles based on bivariate flow karyotyping sequencing data. **C)** Fraction of Hi-C interactions in translocated versus wild-type alleles in Z-138 cells. Deviations from the dashed line (diagonal representing the same fractions in wild-type and translocated chromosomes) represent allele-specific biases. **D)** Density plots of the relative distribution of radial position of chromosome 11 and 14 in the JVM-2 cell line, 0.0 indicates the closest pixel to the lamina and 1.0 the most central position. Dashed lines represent the median. The positioning of chromosome 14 was assessed using the IGH break-apart probe, following the same principle as shown in figure 2D, but then for the IGH locus. **E)** Correlation of non-translocated chromosome positions among MCL, CLL and healthy samples. **F)** Boxplots with the *in silico* modeling-based position of the segments of wild-type or translocated chromosomes 11 and 14. Healthy B cells and CLL contain 200 measurements for each chromosome, while in MCL the two alleles are divided into 100 wild-type and 100 derivative alleles. wt = wild-type, tx = translocated, down = downstream of the breakpoint position, up = upstream of the breakpoint position, ***p-value < 0.001, ns = not significant.

Supplementary figure 3

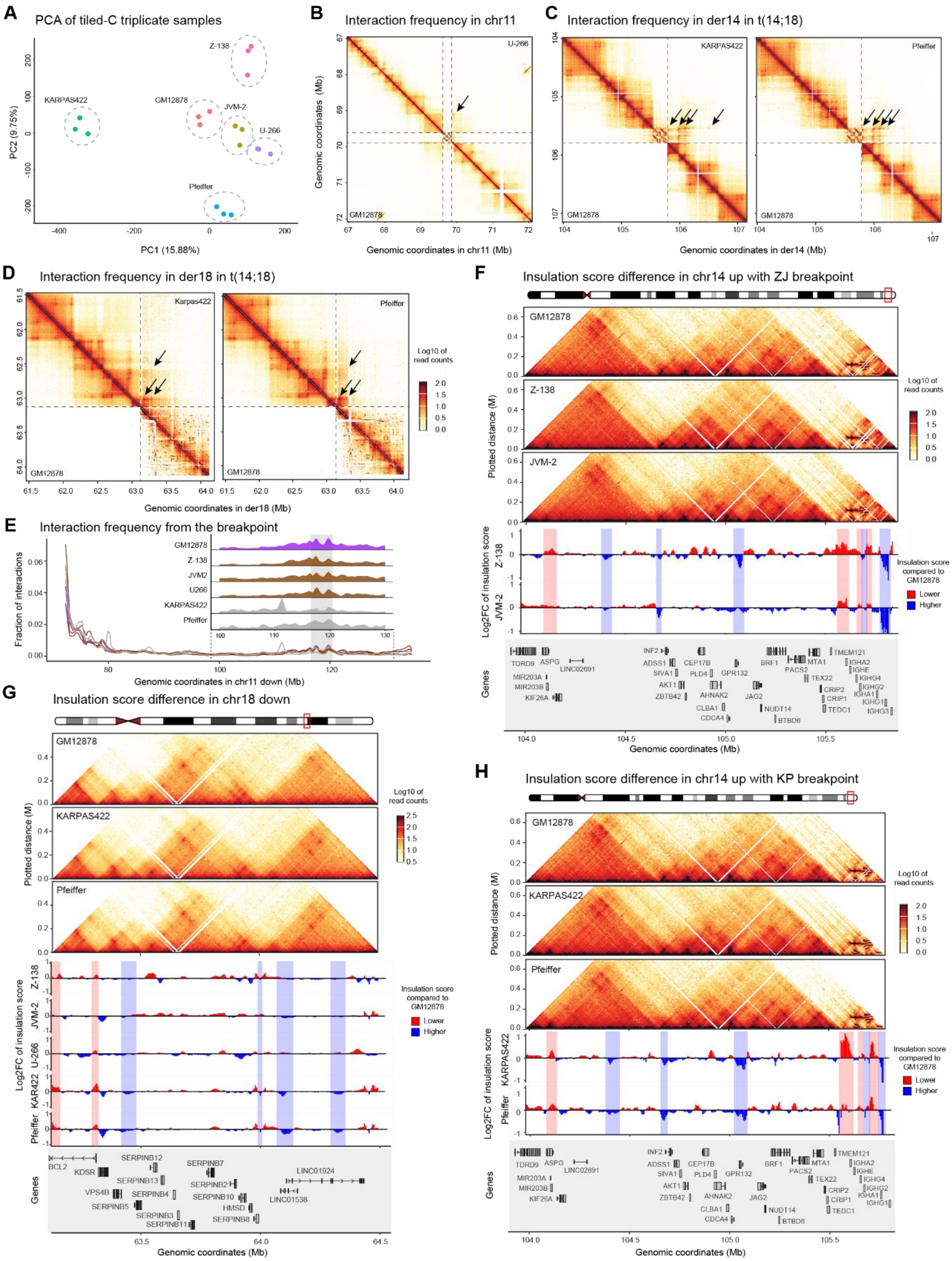

Supplementary Figure 3. Intrachromosomal interaction landscape of translocated chromosomes in MCL. A) PCA of triplicates used in Tiled-C analysis, based on their intrachromosomal interactions within the targeted region. **B)** Tiled-C read count heatmaps in chromosome 11 comparing the U-266 cell line (right-upper triangle) with GM12878 control cells (left-bottom triangle). Dashed lines highlight the region where the IGH enhancer is inserted in chromosome 11 in U-266. Arrows indicate

differences in contact strength from the IGH enhancer insertion to chromosome 11. **C-D)** Same as in B, but depicting derivatives 14 and 18 comparing KARPAS422 and Pfeiffer cell lines carrying the t(14;18) translocation (right-upper triangle) with the control GM12878 (left-bottom triangle). **E)** Interaction frequencies from the breakpoint region within the Tiled-C capture region to the bins outside the capture region in chr11 down. The grey area indicates the CD3 region (chr11: 116,500,000 – 120,500,000). **F-H)** Differences in insulation score on chromosomes chr14 up and chr18 down in the analyzed cell lines compared to GM12878 cells. Heatmaps represent interaction frequencies from Tiled-C read counts in the depicted areas, shown with a red square in the chromosome representation on top. Colors from the line plots indicate a higher (blue) or lower (red) insulation score. der = derivative, wt = wild-type, tx = translocated, down = downstream of the breakpoint position, up = upstream of the breakpoint position, ZJ = Z-138 and JVM-2, KP = KARPAS422 and Pfeiffer.

Supplementary figure 4

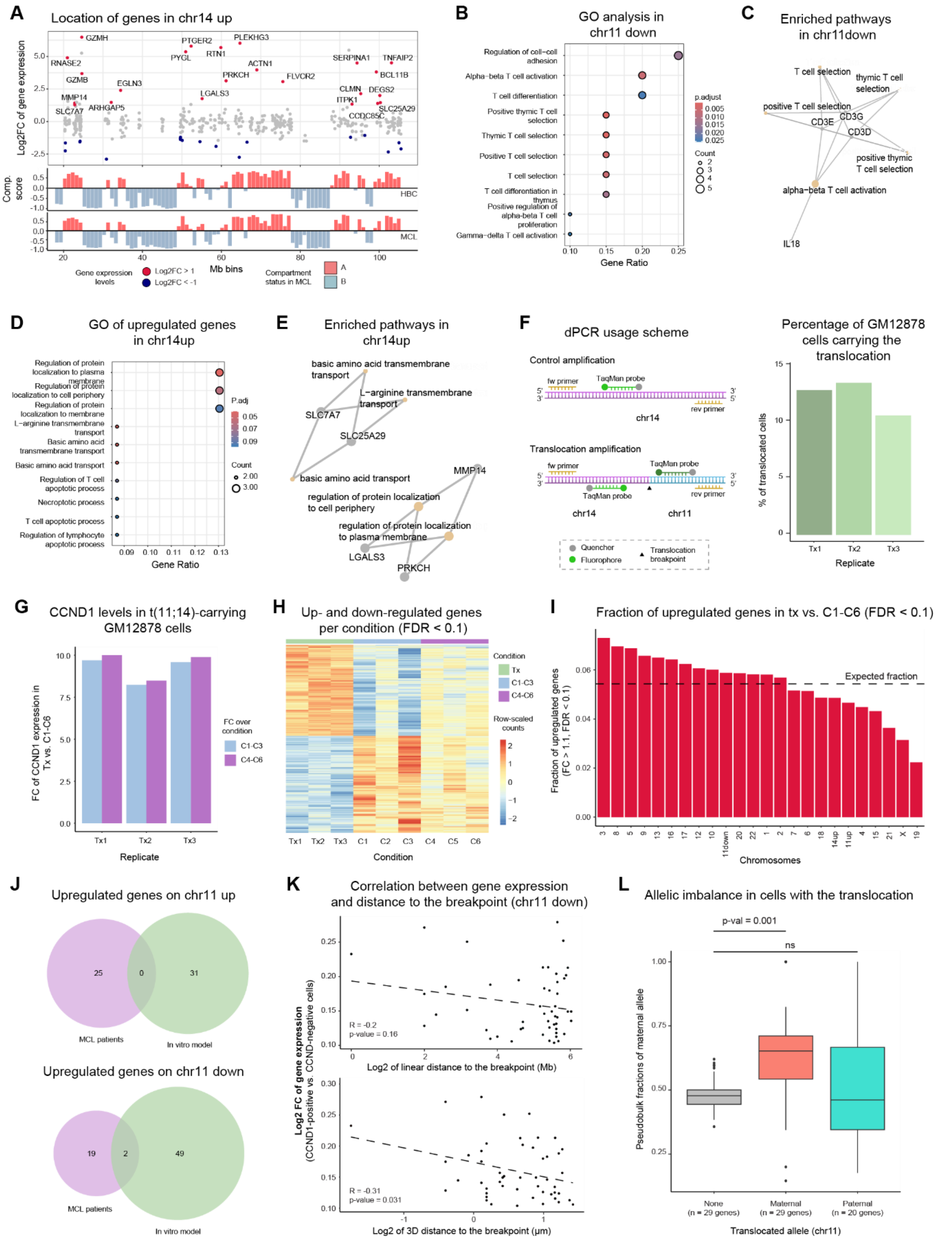

Supplementary Figure 4. Gene expression analysis in cells carrying the t(11;14) translocation. A) Graphic representation of the location of up- and downregulated genes in MCL patients throughout chr14 up. Names for upregulated genes with a log2FC fold change higher than 1 are depicted. Below, the compartment score of each Mb bin in MCL and healthy B cells is represented, whereby blue represents B, and red A compartments. **B-E)** Proportion of genes from the dataset that are linked to upregulated pathways by GO analysis (B and D) and the associated network plots (C and E). Nodes

represent the genes linked to the enriched pathways, and edges indicate the genes that are connected through the same pathways. **F)** Graphical representation (left) and digital PCR results (right) indicating the estimated percentage of cells with translocations after *in vitro* translocation generation in each replicate. **G)** Level of CCND1 upregulation after *in vitro* translocation generation compared to different controls. **H)** VSD-transformed gene expression levels of significantly variable genes in translocated replicates (Tx1-Tx3) compared to controls 1-6 (C1-C6) (FDR < 0.1, absolute log2FC > log2(1.1)). **I)** Fraction of upregulated genes per chromosome in translocated samples compared to C1-C6 controls (FDR < 0.1, FC > 1.1). Dashed line represents the expected fraction of genes. **J)** Overlap between upregulated genes on chromosome 11 in the MCL patients and the *in vitro* translocation models. **K)** Correlation between the gene expression changes in the *in vitro* translocation models in chr11 down (y-axis) and the linear (upper panel) or physical/3D (lower panel, inferred from our in silico models) distance to the breakpoint. CCND1 was excluded for this analysis. **L)** Pseudobulk fraction of transcript counts from the maternal chr11 down in the *in vitro* translocation models, comparing cells with translocations from chromosome 14 to the maternal or paternal alelle of chromosome 11 with cells that do not carry translocations. The total transcript count of maternal and paternal transcripts is set to 1, transcript for which the allele could not be assigned were excluded. down = downstream of the breakpoint position, up = upstream of the breakpoint position. MCL = mantle cell lymphoma, HBC = healthy B cells.
